# Supplementary material for: Identification of Freezing-Responsive microRNAs and Their Targets in Chinese Jujube by Small RNA and Degradome Sequencing
Source: Int J Mol Sci. 2026 Sep 4;27(17):7912. doi: 10.3390/ijms27177912 (PMC13566890; doi:10.3390/ijms27177912)
Supplement: Supplementary file 1 [file ijms-27-07912-s001.zip › Supplementary Material S2/novel_miR_50.pdf]

Provisional ID	: novel_miR_50
Score total	: 103507.3
Score for star read(s)	: 3.9
Score for read counts	: 103501.3
Score for mfe	: 0.5
Score for randfold	: 1.6
Score for cons. seed	:
Total read count	: 203025
Mature read count	: 179050
Loop read count	: 6
Star read count	: 23969

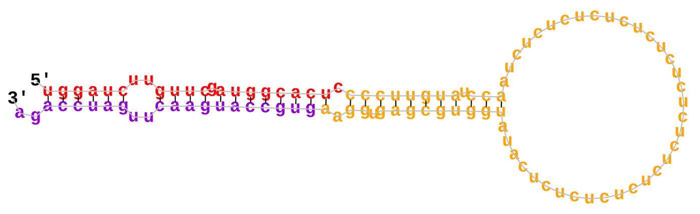

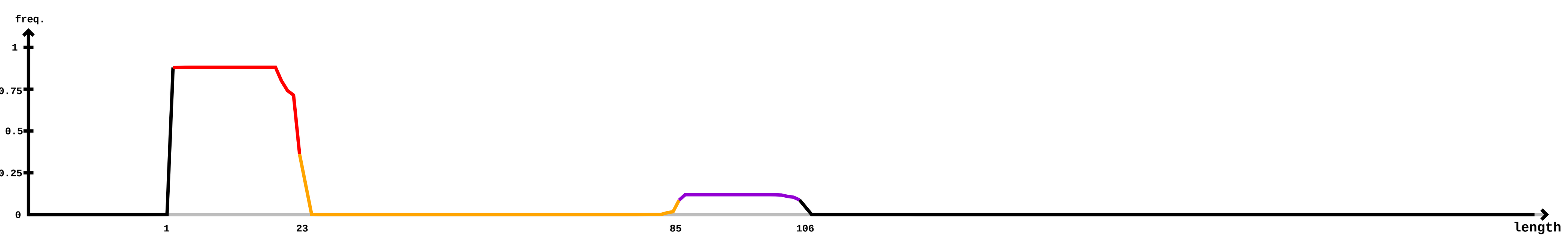

Mature **Star**

```
5'- uccaucuuguucguucacccucuggaucuuguucgauggcacuccccuuguauccaaucucucucucucucucucucucucucucucucucauauggugcgaguggaagugccaugaacuugauccagaaggugcucucgaacaaguuacaccuacuuaauaucuacuucuucuuucuccauuacuauuauuaaugaugguuucuuuuuucugauucucauuuuuaauuuauuuauuuguuuuuuguguuug -3'  obs
    uccaucuuguucguucacccucuggaucuuguucgauggcacuccccuuguauccaaucucucucucucucucucucucucucucucucucauauggugcgaguggaagugccaugaacuugauccagaaggugcucucgaacaaguuacaccuacuuaauaucuacuucuucuuucuccauuacuauuauuaaugaugguuucuuuuuucugauucucauuuuuaauuuauuuauuuguuuuuuguguuug        exp
    .....((((((((..((((.((((((((..((((.((((((((.((((((((.(((..............................))))))))).)).))))))))))))..))))))))).))))....))))))))........................................(((((.(((((..((........))..).)))).)))))........................
```

	reads	mm	sample
...aucuuguucguucacccucugg	2	0	LT-1
......uuguucguucacccucuggaucuuguuc	1	0	LT-1
............uucacccucuggaucuuguu	2	0	LT-2
..................ccucuggaucuuguucgauggc	1	0	JCK-1
..................ccucuggaucuuguucgauggc	1	0	JCK-2
..................ccucuggCucuuguucgauggc	1	1	LT-1
..................ccucuggaucuuguucgauggc	2	0	LT-2
..................ccucuggaucuuguucgauggc	1	0	LT-1
..................ccucuggaucuuguucgauggc	1	0	LT-3
..................ccucuggaucuuguucgauggcacA	1	1	JCK-3
...................cucuggaucuuguuUgau	2	1	JCK-3
...................cucuggaucuuguucgauggc	2	0	JCK-1
...................cucuggaucuuguucgauggc	3	0	JCK-2
...................cucuggaucuuguucgauggc	1	0	JCK-3
...................cucuggaucuuguucgauggc	1	0	LT-1
...................cucuggaucuuguucgauggc	9	0	LT-3
...................cucuggaucuuguucgCuggc	2	1	LT-3
...................Aucuggaucuuguucgauggcacu	1	1	JCK-1
....................ucuggaucuuguucgauggc	3	0	JCK-1
....................ucuggaucuuguucgauggc	1	0	JCK-2
....................ucuggaucuuguucgauggca	2	0	LT-2
....................ucuggaucuuguucgauggca	1	0	JCK-1
....................ucuggaucuuguucgauggca	1	0	JCK-3
....................ucuggaucuuguucgauAgca	1	1	JCK-3
....................ucuggaucuuguucgauggca	4	0	LT-3
....................ucuggaucuuguucgauggcac	1	0	JCK-1
....................ucuggaucuuguucgauggcac	1	0	LT-1
....................ucuggaucuuguucgauggcac	1	0	JCK-2
....................ucuggaucuuguucgauggcacuc	1	0	LT-3
....................ucuggaucuuguucgauggcacuc	2	0	JCK-3
....................ucuggaucuuguucgauggcacuc	3	0	JCK-2
....................ucuggaucuuguucgauggcacuc	1	0	JCK-1
.....................cuggaucuuguucgauggc	3	0	LT-2

Mature **Star**

uccaucuuguucguucacccucuggaucuuguucgauggcacuccccuuguauccaaucucucucucucucucucucucucucucucauauggugcgaguggaagugccaugaacuugauccagaaggugcucucgaacaaguuacaccuacuuaauaucuacuucuucuuucuccauuacuauuauuaaugaugguuucuuuuuucugauucucauuuuuaauuuauuuauuuguuuuuguguuug

cuggaucuuguucgauggc	2	0	LT-1
cuggaucuuguucgauggc	1	0	JCK-1
cuggaucuuguucgauggca	2	0	LT-1
cuggaucuuguucgauggcac	2	0	LCK-3
cuggaucuuguucgauggcacu	7	0	LT-3
cuggaucuuguucgauggcacu	2	0	LT-1
cuggaucuuguucgauggcacu	8	0	JCK-3
cuggaucuuguucgauggcacu	6	0	JCK-1
cuggaucuuguucgauggcacu	16	0	JCK-2
cuggaucuuguucgauggcacu	5	0	LT-2
cuggaucuuguucgauggcacuc	3	0	LT-2
cuggaucuuguucgauggcacuc	2	0	LT-1
cuggaucuuguucgauggcacuc	3	0	JCK-1
cuggaucuuguucgauggcacuc	2	0	JCK-3
cuggaucuuguucgauggcacuU	1	1	LT-3
cuggaucuuguucgauggcacuc	4	0	JCK-2
cuggaucuuguucgauggcacuc	1	0	LT-3
uggaucuuguucgNuggc	2	1	LCK-1
uggaucuuguucgGuggc	1	1	LT-1
uggaucCuguucgauggc	1	1	LCK-3
uggaucuuguucgauggU	7	1	LT-3
uggaucuuguucgCuggc	8	1	LCK-3
uNgaucuuguucgauggc	1	1	JCK-2
ugAaucuuguucgauggc	1	1	LT-2
uggaucuuguucgCuggc	2	1	LCK-1
uggaucuuguucgauggc	2116	0	LT-1
ugAaucuuguucgauggc	1	1	LCK-2
uggaucuuguucgauggA	2	1	LCK-3
uggaucuuguucgaugUc	1	1	LT-2
uggaucCuguucgauggc	1	1	LT-2
uggaucuuguucgauggc	5148	0	LCK-3
uggauAuuguucgauggc	1	1	LT-1
uggaucuuguucgauggG	2	1	LT-3
uggaucuuguucgauggA	6	1	LT-3
uggaucuuguucgauggc	4850	0	LT-2
uggaucuuguucgaCggc	1	1	JCK-2
ugAaucuuguucgauggc	1	1	LCK-3
uggaucuuguucgGuggc	1	1	LCK-1
uggaucuugCucgauggc	1	1	LT-1
uggaucuuguucgauggc	489	0	LCK-2
uUgaucuuguucgauggc	2	1	LT-2
uggaucuuguucgaugCc	1	1	LCK-3
uggaucuuguCcgauggc	3	1	JCK-2
uggaucuuguucgauggU	26	1	LCK-3
uggaucuuguucUauggc	3	1	LT-2
uggaucuuguucgauggA	2	1	LT-2
uggaucuuguucgauggc	261	0	JCK-3
uggaucuuguucgaNggc	6	1	LCK-1
uggaucuuguucgaugAc	4	1	LCK-3
uggGucuuguucgauggc	1	1	LT-1
uggaucuuguuNgauggc	2	1	JCK-1
uggaucuuguucgauggc	186	0	JT-1
uggaucuuguucgauggN	1	1	JCK-3
uggaucuuguucgauggU	3	1	LT-2
uggaucuuguucgauggc	522	0	LCK-1
uAgaucuuguucgauggc	1	1	LT-2
uggaucuuguucgaugAc	3	1	LT-2
uggaucuuguucAauggc	1	1	LCK-3
uggaucuuguucgCuggc	1	1	JT-3
uggaucuuguucgauggc	377	0	JCK-2
uggaucuuguucgauggU	23	1	LCK-1
ugNaucuuguucgauggc	13	1	LT-2
uggaucuuguucgauCgc	1	1	LCK-3
uggaucuuguucgCuggc	5	1	LT-3
uggaucuuguucgauAgc	1	1	LT-2
uggauUuuguucgauggc	1	1	LT-2
uggaucuuguuUgauggc	1	1	LT-1
uggCucuuguucgauggc	9	1	LCK-3
uggaucuuguucgauggc	134	0	JT-2
uAgaucuuguucgauggc	2	1	LT-3

Mature | Star

uccaucuuguucguucacccucuggaucuuguucgauggcacuccccuuguauccaaucucucucucucucucucucucucucucucauauggugcgaguggaagugccaugaacuugauccagaaggugcucucgaacaaguuacaccuacuuaauaucuacuucuucuuucuccauuacuauuauuaaugaugguuucuuuuuucugauucucauuuuuaauuuauuuauuuguuuuuuguguuug

......................ugCaucuuguucgauggc	2	1	LT-2
......................uggaucuuguucUauggc	1	1	LT-1
......................uggaucuuguucgCuggc	1	1	JT-2
......................uggGucuuguucgauggc	1	1	LCK-3
......................uggCucuuguucgauggc	2	1	LT-3
......................uAgaucuuguucgauggc	1	1	LCK-2
......................uggaucuuguucgauggU	22	1	LCK-2
......................uggaucuuguucgauggc	1716	0	LT-3
......................uggCucuuguucgauggc	2	1	LT-1
......................uggaucuugCucgauggc	1	1	JCK-2
......................uAgaucuuguucgauggc	5	1	LCK-3
......................uggaucuuguucgCuggc	1	1	LCK-2
......................uggaucuuguucgauggU	1	1	JCK-3
......................uggaucuuAuucgauggc	1	1	LCK-3
......................uggaucuugCucgauggc	1	1	LT-3
......................uggaucuuguucgauggc	182	0	JT-3
......................uggaucuugCucgauggc	2	1	LT-2
......................uggCucuuguucgauggc	37	1	LT-2
......................uggaucuuguucgCuggc	7	1	LT-1
......................uggaucuuguucgauAgc	1	1	LCK-3
......................uAgaucuuguucgauggc	1	1	LT-1
......................uggCucuuguucgauggc	1	1	JCK-2
......................ugAaucuuguucgauggc	1	1	LT-3
......................Cggaucuuguucgauggc	1	1	LCK-3
......................uggaucuuguucgauggA	2	1	JCK-3
......................uggaucuuguucgauggU	5	1	LT-1
......................uggaucuuguucgCuggc	19	1	LT-2
......................uggaucuuguucgauAgc	1	1	JCK-1
......................uggaCcuuguucgauggc	1	1	LT-2
......................ugAaucuuguucgauggc	1	1	LT-1
......................uNgaucuuguucgauggc	1	1	JCK-3
......................uggaucuuguucAauggc	1	1	LT-2
......................uggaucuuguucgauggc	253	0	JCK-1
......................uAgaucuuguucgauggca	2	1	LT-2
......................uggaucuuguucgauggcU	20	1	LCK-2
......................uggaucuuguucgGuggca	1	1	LCK-1
......................uggaucuuguucgauggca	306	0	JCK-3
......................uggaucuuguucgCuggca	3	1	LCK-2
......................uggaucuuguucgCuggca	1	1	LT-3
......................uggaucuuguGcgauggca	1	1	JCK-1
......................ugAaucuuguucgauggca	1	1	LCK-2
......................uggaucuuguucgauggcG	1	1	LT-3
......................uggaucuuguucgCuggca	2	1	LCK-3
......................uggCucuuguucgauggca	7	1	LCK-2
......................uggaucuuguucgauggAa	2	1	LT-1
......................uggaucuuguCcgauggca	1	1	LCK-3
......................uggaucuuguucgauggca	281	0	JT-3
......................uggaucuuAuucgauggca	1	1	LCK-2
......................uggaucuuguuAgauggca	1	1	LCK-2
......................uggaucuuguucgauggcU	24	1	LCK-1
......................uggaucuuguucgauggca	1539	0	LCK-1
......................uggCucuuguucgauggca	7	1	LCK-3
......................uggCucuuguucgauggca	2	1	JCK-1
......................uggaucuuguucgauggcG	8	1	LCK-3
......................uggaucuuguucgauggca	1208	0	LT-3
......................uggaucuuguucgauggcG	1	1	JCK-1
......................uggaucuuguucgauggcU	21	1	LT-2
......................uggaucuuguucgauggca	411	0	JCK-2
......................uggaucuuguucAauggca	1	1	LCK-1
......................uggaucuuguucgCuggca	10	1	LT-2
......................uggaucuuguucgauggcC	1	1	LCK-1
......................uggaucuuguucgauggcU	11	1	LT-1
......................uggaucuuguucgauggcC	1	1	LT-1
......................uggGucuuguucgauggca	1	1	LCK-1
......................uggaucuuguucgaCggca	1	1	JT-1
......................uggaucuuguucgauggca	1462	0	LCK-2
......................ugAaucuuguucgauggca	2	1	LCK-1
......................uggauNuuguucgauggca	2	1	LCK-1
......................uggaucuuguucgauggUa	1	1	LCK-3
......................uggaucuuguucgauggca	377	0	JT-1

Mature **Star**

uccaucuuguucguucacccucuggaucuuguucgauggcacuccccuuguauccaaucucucucucucucucucucucucucucucucauauggugcgaguggaagugccaugaacuugauccagaaggugcucucgaacaaguuacaccuacuuaauaucuacuucuucuuucuccauuacuauuauuaaugaugguuucuuuuuucugauucucauuuuaauuuauuuauuuguuuuuguguuug

......................Cggaucuuguucgauggca...	1	1	JCK-2
......................ugNaucuuguucgauggca...	1	1	LT-2
......................uggaucuuAuucgauggca...	1	1	LT-1
......................uAgaucuuguucgauggca...	1	1	LCK-3
......................ugAaucuuguucgauggca...	1	1	LCK-3
......................uggaucuuguucgauggca...	2503	0	LCK-3
......................uggGucuuguucgauggca...	1	1	LCK-3
......................uggaucuuguucgaGggca...	1	1	LCK-1
......................uggaucuuguucgauAgca...	2	1	LCK-2
......................uggCucuuguucgauggca...	3	1	LCK-1
......................uggaucuuguucgauggcU...	8	1	JCK-2
......................uggaucuuguucUauggca...	3	1	LT-2
......................uggaucuuguucgCuggca...	1	1	JCK-1
......................uggaucuuguucUauggca...	6	1	LCK-3
......................uggaucuuAuucgauggca...	1	1	LT-2
......................uggaucuuguucgauggcG...	2	1	JT-3
......................uggaucuuguuUgauggca...	1	1	JT-1
......................uggaucuuguucgauggcG...	1	1	LT-1
......................uggaucuuguucgauggcU...	24	1	LT-3
......................uggaucuuguucgauggcU...	1	1	JCK-3
......................uggCucuuguucgauggca...	10	1	LT-2
......................uggaucuuguucgauAgca...	1	1	JCK-3
......................ugAaucuuguucgauggca...	1	1	LT-2
......................uggaucuuguucgauggcU...	7	1	JT-3
......................uggaucuuAuucgauggca...	1	1	LT-3
......................uggCucuuguucgauggca...	1	1	LT-1
......................uggaucuuguucNauggca...	1	1	LCK-1
......................uggaucuuguucgauggcC...	2	1	LCK-3
......................uggaucuuguucgauggca...	259	0	JCK-1
......................uggNucuuguucgauggca...	1	1	JCK-2
......................uggaucuCguucgauggca...	1	1	LT-2
......................uggaucuuUuucgauggca...	1	1	LT-2
......................uggCucuuguucgauggca...	2	1	LT-3
......................uggaucuuguucgauggcC...	1	1	LT-3
......................uggaucuuguucgauggca...	1906	0	LT-2
......................uggaucuuguucgauggcU...	13	1	LCK-3
......................uAgaucuuguucgauggca...	1	1	LCK-2
......................uggaucuuguucgNuggca...	7	1	LCK-1
......................uggaucuuguucgauggcU...	2	1	JT-2
......................uggaucuuguucgauAgca...	1	1	LT-2
......................uggaucuuguucUauggca...	1	1	LT-3
......................uggaucuuguucgauggcC...	5	1	LT-2
......................uggaucuuguucgCuggca...	1	1	JCK-2
......................uggaucuuguucgauggcC...	1	1	JT-1
......................uggaucuuguucgauggca...	1101	0	LT-1
......................uggaucuuguucgaugAca...	2	1	LT-1
......................uggaucuCguucgauggca...	1	1	LCK-3
......................uAgaucuuguucgauggca...	2	1	LT-3
......................uggaucuuguucgaNggca...	2	1	LCK-1
......................uggaucuuguucgauAgca...	4	1	JCK-1
......................uggaucuuguucgauAgca...	4	1	JCK-2
......................uggaucuuguucgGuggca...	1	1	LT-3
......................uggaucuuguucgCuggca...	2	1	LCK-1
......................uggaucuuguucgauggca...	182	0	JT-2
......................uggaucuuguucgauggcCc...	1	1	LT-3
......................uggCucuuguucgauggcac...	3	1	LT-2
......................uggaucuuguuUgauggcac...	1	1	LT-1
......................uggaucuuguucgCuggcac...	1	1	LT-1
......................uggCucuuguucgauggcac...	3	1	LCK-3
......................uggaucuuguucgauggcac...	427	0	LCK-1
......................uggaucuuguucgauggcac...	108	0	JT-1
......................uggaucuuguucgauggcCc...	1	1	LT-2
......................ugAaucuuguucgauggcac...	1	1	JCK-3
......................uggaucuuguucgaugAcac...	1	1	LT-2
......................uggaucuuguucgCuggcac...	1	1	LCK-3
......................uggCucuuguucgauggcac...	1	1	LT-1
......................uggaucuuguucgaNggcac...	1	1	LCK-1
......................uggaucuuguucgauggcac...	118	0	JCK-3
......................uggaucuuguucgauggcac...	19	0	JT-2
......................ugNaucuuguucgauggcac...	4	1	LT-2

Mature (red): uggaucuuguucgauggcacuc

Star (purple): gugccaugaacuugauccaga

uccaucuuguucguucacccucuggaucuuguucgauggcacuccccuuguauccaaucucucucucucucucucucucucucucucucauauggugcgaguggaagugccaugaacuugauccagaaggugcucucgaacaaguuacaccuacuuaauaucuacuucuucuuucuccauuacuauuauuaaugaugguuucuuuuuucugauucucauuuuuaauuuauuuauuuguuuuuuguguuug

uggaucuCguucgauggcac	1	1	LT-2
uggaucuuguucgauggcaU	12	1	LCK-3
uggCucuuguucgauggcac	1	1	JCK-1
uggaucuuguucgauggcGc	1	1	LT-2
uggaucuuguucgauggcac	40	0	JT-3
uggaucuuguucgCuggcac	3	1	LT-2
uggaucuuguucgCuggcac	2	1	LT-3
uggaucuuguucgauggcCc	1	1	LCK-3
uggaucuuguucgauggcaU	3	1	JT-3
uggaucuuguucgauAgcac	3	1	JCK-2
uggaucuuguucgauggcaU	26	1	LT-3
Cggaucuuguucgauggcac	1	1	LT-3
uggaucuuguucgauggcaU	3	1	JCK-1
uggaucuuguucgauggcac	183	0	JCK-2
uggaCcuuguucgauggcac	1	1	LT-2
uggaucuuguucgauggcUc	1	1	LT-2
uggaucuuguucgauggcaU	4	1	JCK-3
uggaucuuguucgaGggcac	1	1	LCK-1
uggaucuuguucgauggcac	122	0	JCK-1
uggaucuuguucgauggcac	704	0	LT-3
uggaucCuguucgauggcac	1	1	LT-3
uggaucuuguucgauggcac	1673	0	LCK-3
uAgaucuuguucgauggcac	1	1	LCK-3
uggaucuuguucgCuggcac	1	1	JCK-1
uggaucuuCuucgauggcac	1	1	LT-2
uggaucuuguucgaCggcac	1	1	JCK-2
uggaucuuguucUauggcac	1	1	JT-1
uggaucuuguucgauggcac	571	0	LT-1
uggaucuuguucgauAgcac	1	1	LT-2
uggaucuuguucgauggcaU	13	1	LT-2
uggaucuuguucgauggcaA	7	1	LT-2
uggCucuuguucgauggcac	1	1	JCK-3
uggaucuuguucgCuggcac	1	1	LCK-1
uggaucuuguucgauAgcac	1	1	JCK-1
uggaucuuguucgauAgcac	3	1	JCK-3
uggaucuuguucgauggcaA	2	1	LT-1
uggaucuuguucgauggcac	916	0	LT-2
uggaucuuguucgauggcaU	1	1	JT-2
uggaucuuguucgauggcac	427	0	LCK-2
uAgaucuuguucgauggcac	1	1	LT-2
uggCucuuguucgauggcac	1	1	LT-3
uggaucuuguucgNuggcac	2	1	LCK-1
uggaucuuguucgauggcCc	1	1	LT-1
uggaucuuguuUgauggcac	1	1	LCK-3
uggaucuuguucgaCggcac	1	1	LT-1
uggaucuuguucgauggcaU	13	1	LT-1
uggaucuuguucgaCggcac	1	1	LT-2
uggaucuuguucgauggcUc	6	1	LCK-3
uggaucuuguucgauggcaG	1	1	JCK-1
uggNucuuguucgauggcac	1	1	LCK-1
uggaucuuguucgauggUacu	6	1	JCK-2
uggaucuuguucgauggUacu	1	1	LT-3
uggaucuugCucgauggcacu	1	1	LT-1
ugAaucuuguucgauggcacu	3	1	LT-1
uggaucuuguucgauggcCcu	5	1	LCK-3
uggaucuuguucgCuggcacu	20	1	LT-2
uggCucuuguucgauggcacu	24	1	JCK-1
uggaucCuguucgauggcacu	1	1	LCK-3
uggaucCuguucgauggcacu	3	1	JCK-1
uggaucCuguucgauggcacu	1	1	LT-3
uggaucuuguucgauggcaUu	10	1	LT-1
uggaucuuguucgauggcacu	181	0	JT-2
uggCucuuguucgauggcacu	37	1	JCK-2
uggaucuuguucgCuggcacu	18	1	LT-3
uggaucuCguucgauggcacu	2	1	JCK-2
uAgaucuuguucgauggcacu	2	1	JCK-3
uggaucuuguucAauggcacu	2	1	JCK-3
uggaucuuguucgGuggcacu	1	1	JCK-1
Cggaucuuguucgauggcacu	1	1	LT-2
uggaucuuguucgauggcacu	288	0	JT-3

```
                      Mature                                                                    Star
uccaucuuguucguucacccucuggaucuuguucgauggcacuccccuuguauccaaucucucucucucucucucucucucucucucauauggugcgaguggaagugccaugaacuugauccagaaggugcucucgaacaaguuacaccuacuuaaauaucuacuucuucuuucuccauuacuauuauuaaugaugguuucuuuuuucugauucucauuuuuaauuuauuuauuuguuuuuguguuug
......................uggaucuuguucgauggAacu..............................................................................................................................................................................   1      1   JCK-1
......................uggaucuuguucgauggcaUu..............................................................................................................................................................................   23     1   LCK-1
......................uggaucuuguucgauggcaAu..............................................................................................................................................................................   8      1   JCK-2
......................uggaucuuguucgauggcacu..............................................................................................................................................................................   7444   0   LT-3
......................uAgaucuuguucgauggcacu..............................................................................................................................................................................   3      1   LCK-3
......................uggaucuuguucgaugAcacu..............................................................................................................................................................................   9      1   JCK-2
......................uggaucuuguucgauggcacG..............................................................................................................................................................................   5      1   LT-1
......................uggaucuuguucgauggcacA..............................................................................................................................................................................   12     1   LT-1
......................uggaucuuguuUgauggcacu..............................................................................................................................................................................   1      1   LCK-2
......................uggaucuuguucgCuggcacu..............................................................................................................................................................................   1      1   JT-2
......................uggaucCuguucgauggcacu..............................................................................................................................................................................   4      1   JCK-3
......................uggaucuuguucgauggcCcu..............................................................................................................................................................................   6      1   JCK-3
......................uggaucuuguucgauggcacC..............................................................................................................................................................................   7      1   LT-2
......................uggaucuuguucgauggcacG..............................................................................................................................................................................   7      1   JCK-1
......................uggaucuuguucgCuggcacu..............................................................................................................................................................................   12     1   JCK-1
......................uggaucuuguucgauggcacG..............................................................................................................................................................................   1      1   LCK-1
......................uggaucuuguuNgauggcacu..............................................................................................................................................................................   21     1   JCK-1
......................uggaucuuguucUauggcacu..............................................................................................................................................................................   1      1   JCK-2
......................uggaucuuguCcgauggcacu..............................................................................................................................................................................   3      1   JCK-2
......................uNgaucuuguucgauggcacu..............................................................................................................................................................................   1      1   LCK-1
......................uggaucuuguucgGuggcacu..............................................................................................................................................................................   1      1   JCK-3
......................uggaucuuguuUgauggcacu..............................................................................................................................................................................   2      1   LT-1
......................uggaucuuguucgauggcacu..............................................................................................................................................................................   5446   0   LT-2
......................ugAaucuuguucgauggcacu..............................................................................................................................................................................   3      1   LCK-3
......................uggaucuuguucgauAgcacu..............................................................................................................................................................................   26     1   JCK-2
......................uggaucuuguucgauggUacu..............................................................................................................................................................................   5      1   JCK-3
......................uggaucuuguucgauggcacC..............................................................................................................................................................................   7      1   JCK-2
......................uNgaucuuguucgauggcacu..............................................................................................................................................................................   1      1   LCK-3
......................uggaucuuguucgauAgcacu..............................................................................................................................................................................   11     1   JCK-3
......................uggaucuuNuucgauggcacu..............................................................................................................................................................................   1      1   JCK-3
......................uggaCcuuguucgauggcacu..............................................................................................................................................................................   2      1   JCK-2
......................uUgaucuuguucgauggcacu..............................................................................................................................................................................   2      1   LT-2
......................uggaucuuguucgauggcacC..............................................................................................................................................................................   11     1   LCK-3
......................uggaucuuUuucgauggcacu..............................................................................................................................................................................   3      1   JCK-3
......................uggCucuuguucgauggcacu..............................................................................................................................................................................   25     1   LT-3
......................uggaucuuguucgauggcacC..............................................................................................................................................................................   12     1   JCK-3
......................uggaCcuuguucgauggcacu..............................................................................................................................................................................   1      1   LT-2
......................uggaucuuguucgauAgcacu..............................................................................................................................................................................   2      1   LT-1
......................uggaucuuguucAauggcacu..............................................................................................................................................................................   3      1   LT-2
......................uggaucuuguucgauggcGcu..............................................................................................................................................................................   1      1   LT-2
......................uggaucuuguucgauAgcacu..............................................................................................................................................................................   2      1   LT-3
......................uggaucuuguucgaCggcacu..............................................................................................................................................................................   1      1   LT-1
......................uggaucuuguucgauggcacA..............................................................................................................................................................................   100    1   LCK-3
......................uggaucuuguucgCuggcacu..............................................................................................................................................................................   11     1   LCK-3
......................uggaucuuguucgauggcacG..............................................................................................................................................................................   1      1   LCK-3
......................uggaucuuguucgaCggcacu..............................................................................................................................................................................   1      1   LCK-3
......................uggaucuuguucgauggcacG..............................................................................................................................................................................   9      1   LT-3
......................uggaucuuguucgauggcacu..............................................................................................................................................................................   9876   0   JCK-1
......................uggCucuuguucgauggcacu..............................................................................................................................................................................   5      1   LCK-2
......................uggaucuuguucNauggcacu..............................................................................................................................................................................   1      1   LCK-1
......................uggaucuuguucUauggcacu..............................................................................................................................................................................   4      1   JCK-3
......................uggaucCuguucgauggcacu..............................................................................................................................................................................   6      1   JCK-2
......................uggaucuuguucgaGggcacu..............................................................................................................................................................................   2      1   LCK-1
......................uggaucuuguucgauggcCcu..............................................................................................................................................................................   2      1   LCK-1
......................ugAaucuuguucgauggcacu..............................................................................................................................................................................   3      1   JCK-3
......................uggaucuuguucgaCggcacu..............................................................................................................................................................................   5      1   JCK-2
......................uggaucuuguucgauggcacG..............................................................................................................................................................................   2      1   LT-2
......................uggaucuuAuucgauggcacu..............................................................................................................................................................................   1      1   JCK-2
......................uggaucuuguucgauggAacu..............................................................................................................................................................................   1      1   LCK-3
......................uggaucuuguucgaugUcacu..............................................................................................................................................................................   1      1   JCK-1
......................uggaucuuguucgauggUacu..............................................................................................................................................................................   2      1   JCK-1
......................uggaucuuguucgauggcaUu..............................................................................................................................................................................   10     1   JCK-1
......................uggaucuuguCcgauggcacu..............................................................................................................................................................................   2      1   JCK-1
......................uggaucuuguucCauggcacu..............................................................................................................................................................................   1      1   JCK-3
......................uggaucuuguucgauggGacu..............................................................................................................................................................................   4      1   JCK-1
......................uggaucuuguucgaugAcacu..............................................................................................................................................................................   4      1   JCK-3
......................uggaucuuAuucgauggcacu..............................................................................................................................................................................   3      1   LT-2
......................uggauNuuguucgauggcacu..............................................................................................................................................................................   3      1   LCK-1
......................uggaucuuguucgaugAcacu..............................................................................................................................................................................   4      1   JCK-1
......................uggaucuuAuucgauggcacu..............................................................................................................................................................................   2      1   LCK-3
```

Mature Star

uccaucuuguucguucacccucuggaucuuguucgauggcacuccccuuguauccaaucucucucucucucucucucucucucucucucucucauauggugcgaguggaagugccaugaacuugauccagaaggugcucucgaacaaguuacaccuacuuaauaucuacuucuucuuucuccauuacuauuauuaaugaugguuucuuuuuucugauucucauuuuuaauuuauuuauuuguuuuuuguguuug			
......................uggaucuuUuucgauggcacu..	1	1	LT-1
......................uggauUuuguucgauggcacu..	3	1	JCK-2
......................Cggaucuuguucgauggcacu..	1	1	LT-1
......................uggaucuuguuAgauggcacu..	2	1	JCK-1
......................uggaucuuguucgauggcacA..	31	1	JCK-2
......................uggaucuuguucgauggcacA..	25	1	JCK-3
......................uggaucuuguucgCuggcacu..	33	1	JCK-2
......................uggaucuuguucgauggcaUu..	10	1	LCK-3
......................uggCucuuguucgauggcacu..	22	1	JCK-3
......................uUgaucuuguucgauggcacu..	3	1	JCK-2
......................uggaucuuguuUgauggcacu..	4	1	LT-3
......................uggaucuuguucAauggcacu..	12	1	JCK-2
......................uggaucuuguucgauggcGcu..	1	1	LCK-3
......................Cggaucuuguucgauggcacu..	4	1	LCK-3
......................uggaucuugCucgauggcacu..	1	1	JCK-1
......................uNgaucuuguucgauggcacu..	6	1	JCK-3
......................uggaucuuguucgauggcCcu..	6	1	LT-1
......................uggaucuuguucgauggcaUu..	21	1	LCK-2
......................uggaucuuguucgauggcacu..	1957	0	LCK-2
......................uggaucuuguucgauggcaAu..	1	1	LCK-3
......................uggaucuuguucgaugUcacu..	1	1	LT-3
......................uggaucuuguucgaCggcacu..	2	1	JCK-1
......................uggaucuuguuUgauggcacu..	9	1	JCK-3
......................uggaucuuguucgauggcacA..	34	1	LCK-1
......................uggaucuuguucgauAgcacu..	12	1	JCK-1
......................uggaucuuguuUgauggcacu..	1	1	JCK-1
......................uAgaucuuguucgauggcacu..	1	1	LT-1
......................uggaucuuguucgaCggcacu..	1	1	LCK-2
......................uggaucuuAuucgauggcacu..	1	1	JCK-3
......................uggauUuuguucgauggcacu..	1	1	LT-2
......................uggCucuuguucgauggcacu..	15	1	LT-1
......................uggaucuuguuAgauggcacu..	1	1	JCK-3
......................uggaucuuguucgNuggcacu..	12	1	LCK-1
......................uggaucuuguucgauggcaUu..	12	1	JCK-2
......................uggaucuuguucgauggcacu..	6606	0	LCK-3
......................uggaucuuguucgaugAcacu..	3	1	LCK-3
......................uggaucuuguucAauggcacu..	3	1	JCK-1
......................uAgaucuuguucgauggcacu..	2	1	JCK-2
......................ugAaucuuguucgauggcacu..	8	1	JCK-2
......................Gggaucuuguucgauggcacu..	1	1	LT-2
......................uggCucuuguucgauggcacu..	6	1	LCK-1
......................uggaucuuguucgauggcCcu..	2	1	LCK-2
......................uggaucuuguucgauggcNcu..	6	1	JCK-1
......................uggaucuuguucgauggcaNu..	8	1	JCK-1
......................uggaucuuguucgauggcacG..	7	1	JCK-2
......................uggaucuuguucgCuggcacu..	1	1	LCK-1
......................uggaucuuguucgauggcUcu..	1	1	JT-2
......................uggaucuuguucAauggcacu..	3	1	LT-3
......................uggaucuuguucgauggcaUu..	14	1	LT-3
......................uAgaucuuguucgauggcacu..	1	1	JCK-1
......................uggCucuuguucgauggcacu..	12	1	LCK-3
......................uggaucuuguucgauggcCcu..	10	1	JCK-1
......................uggCucuuguucgauggcacu..	1	1	JT-1
......................uggaucuuguucgaNggcacu..	5	1	LCK-1
......................uggaucuuguucUauggcacu..	4	1	JCK-1
......................uggaucuuguucgaugAcacu..	5	1	LT-3
......................uAgaucuuguucgauggcacu..	4	1	LT-3
......................uggaucuuguucgCuggcacu..	10	1	LT-1
......................uggaucuuguucgauggcacC..	15	1	LT-1
......................uggaucuuguucgCuggcacu..	3	1	LCK-2
......................uggaucuugCucgauggcacu..	2	1	JCK-2
......................uggaucuuguucgauggcCcu..	21	1	JCK-2
......................uggaucuuguucgauggcCcu..	12	1	LT-2
......................uggaucuuguucgaugUcacu..	2	1	JCK-2
......................ugNaucuuguucgauggcacu..	1	1	LCK-1
......................uggaucuuAuucgauggcacu..	1	1	LT-3
......................uggaucuuguGcgauggcacu..	2	1	JCK-1
......................Gggaucuuguucgauggcacu..	1	1	LT-3
......................uggaucuuguucgauggcCcu..	1	1	JT-1
......................uggaucuuguucgauggcCcu..	3	1	LT-3

```
                      Mature                                                                                Star
uccaucuuguucguucacccucuggaucuuguucgauggcacuccccuuguauccaaucucucucucucucucucucucucucucucucauauggugcgaguggaagugccaugaacuugauccagaaggugcucucgaacaaguuacaccuacuuaauaucuacuucuucuuucuccauuacuauuauuaaugaugguuucuuuuuucugauucucauuuuuaauuuauuuauuuguuuuuguguuug
......................uggaucuuguucgauggcacA........................................................................................................................................................................................................ 28     1    LT-2
......................uggaucuuguucgUuggcacu........................................................................................................................................................................................................ 1      1    LT-2
......................Cggaucuuguucgauggcacu........................................................................................................................................................................................................ 4      1    JCK-2
......................uggaucuuguucAauggcacu........................................................................................................................................................................................................ 1      1    LT-1
......................uggaucuuguucgaugAcacu........................................................................................................................................................................................................ 8      1    LT-1
......................uggaucuuguucgauggcacN........................................................................................................................................................................................................ 1      1    JT-2
......................uAgaucuuguucgauggcacu........................................................................................................................................................................................................ 1      1    LCK-2
......................uggaucuuguuUgauggcacu........................................................................................................................................................................................................ 8      1    JCK-2
......................uNgaucuuguucgauggcacu........................................................................................................................................................................................................ 10     1    JCK-1
......................Cggaucuuguucgauggcacu........................................................................................................................................................................................................ 1      1    JCK-3
......................ugNaucuuguucgauggcacu........................................................................................................................................................................................................ 8      1    LT-2
......................uggaucuuguCcgauggcacu........................................................................................................................................................................................................ 1      1    LT-2
......................uggaucuuguucgauggcacu........................................................................................................................................................................................................ 712    0    JT-1
......................uggaucuuguucgauggcacu........................................................................................................................................................................................................ 1998   0    LCK-1
......................uggaucuuguucgauggcaAu........................................................................................................................................................................................................ 8      1    LT-2
......................uggaucuuguCcgauggcacu........................................................................................................................................................................................................ 1      1    LT-3
......................uggaucuuguucgauggcUcu........................................................................................................................................................................................................ 1      1    LT-1
......................Cggaucuuguucgauggcacu........................................................................................................................................................................................................ 1      1    JT-1
......................uggaucuuguucgauggcacu........................................................................................................................................................................................................ 12438  0    JCK-3
......................uggaucuuguucgauggcacA........................................................................................................................................................................................................ 61     1    LT-3
......................uggaucuuguucgauggcUcu........................................................................................................................................................................................................ 1      1    LT-3
......................uggaucuuguucgauggcacA........................................................................................................................................................................................................ 30     1    JCK-1
......................uggGucuuguucgauggcacu........................................................................................................................................................................................................ 5      1    JCK-1
......................uggNucuuguucgauggcacu........................................................................................................................................................................................................ 10     1    JCK-1
......................uggaucuuguucgauggNacu........................................................................................................................................................................................................ 1      1    JCK-3
......................uggaucuuguucgaugAcacu........................................................................................................................................................................................................ 1      1    JT-3
......................uggaucuuguucgauggcacG........................................................................................................................................................................................................ 6      1    JCK-3
......................uggaucAuguucgauggcacu........................................................................................................................................................................................................ 2      1    JCK-2
......................uggaucuuguucgauggcaAu........................................................................................................................................................................................................ 2      1    LT-1
......................uggaucuuguucgCuggcacu........................................................................................................................................................................................................ 4      1    JT-1
......................uggaucuuguucgauggcaAu........................................................................................................................................................................................................ 3      1    JCK-1
......................uggaucuuguucgauggcGcu........................................................................................................................................................................................................ 1      1    JCK-3
......................uggaucuuguucgauggcaAu........................................................................................................................................................................................................ 1      1    JCK-3
......................uggaCcuuguucgauggcacu........................................................................................................................................................................................................ 2      1    JCK-3
......................Gggaucuuguucgauggcacu........................................................................................................................................................................................................ 2      1    JCK-2
......................uggaucuuUuucgauggcacu........................................................................................................................................................................................................ 2      1    LT-3
......................uggGucuuguucgauggcacu........................................................................................................................................................................................................ 1      1    JCK-2
......................uggaucuuguucgauggcaUu........................................................................................................................................................................................................ 8      1    JCK-3
......................Cggaucuuguucgauggcacu........................................................................................................................................................................................................ 1      1    LT-3
......................uggaucuuguucgauggcacC........................................................................................................................................................................................................ 3      1    JCK-1
......................uggaucuuguucgauggcacA........................................................................................................................................................................................................ 24     1    LCK-2
......................uggaucuuguucgauggcaGu........................................................................................................................................................................................................ 1      1    JCK-1
......................uggaucuuguucgCuggcacu........................................................................................................................................................................................................ 18     1    JCK-3
......................uggaucuuguucgauggcacN........................................................................................................................................................................................................ 1      1    JCK-1
......................uggaucuuguuGgauggcacu........................................................................................................................................................................................................ 2      1    JCK-1
......................uggNucuuguucgauggcacu........................................................................................................................................................................................................ 1      1    LCK-1
......................uUgaucuuguucgauggcacu........................................................................................................................................................................................................ 2      1    JCK-3
......................uNgaucuuguucgauggcacu........................................................................................................................................................................................................ 18     1    JCK-2
......................uCgaucuuguucgauggcacu........................................................................................................................................................................................................ 1      1    JCK-2
......................ugAaucuuguucgauggcacu........................................................................................................................................................................................................ 4      1    LT-2
......................ugCaucuuguucgauggcacu........................................................................................................................................................................................................ 1      1    LT-2
......................uggaCcuuguucgauggcacu........................................................................................................................................................................................................ 1      1    LCK-1
......................uggaucuuguucgauggcacC........................................................................................................................................................................................................ 15     1    LT-3
......................uggaCcuuguucgauggcacu........................................................................................................................................................................................................ 1      1    LCK-2
......................uggaucuuguucgauggcGcu........................................................................................................................................................................................................ 1      1    JCK-2
......................uggGucuuguucgauggcacu........................................................................................................................................................................................................ 1      1    LT-2
......................uggaucuuguucgCuggcacu........................................................................................................................................................................................................ 2      1    JT-3
......................uggaucuuguucgaCggcacu........................................................................................................................................................................................................ 1      1    LT-2
......................uAgaucuuguucgauggcacu........................................................................................................................................................................................................ 4      1    LT-2
......................uggCucuuguucgauggcacu........................................................................................................................................................................................................ 53     1    LT-2
......................uggaucuuguucgauggcacu........................................................................................................................................................................................................ 17137  0    JCK-2
......................uggaucuuguucAauggcacu........................................................................................................................................................................................................ 1      1    LCK-3
......................uggaucuCguucgauggcacu........................................................................................................................................................................................................ 1      1    JCK-1
......................uggaucuCguucgauggcacu........................................................................................................................................................................................................ 1      1    LT-1
......................uggaucuuguucgauggcaUu........................................................................................................................................................................................................ 8      1    LT-2
......................uggGucuuguucgauggcacu........................................................................................................................................................................................................ 2      1    LT-3
......................ugAaucuuguucgauggcacu........................................................................................................................................................................................................ 1      1    JCK-1
......................uggaCcuuguucgauggcacu........................................................................................................................................................................................................ 2      1    LT-1
......................ugAaucuuguucgauggcacu........................................................................................................................................................................................................ 2      1    LT-3
......................uggaucuuguucgauggcacu........................................................................................................................................................................................................ 6201   0    LT-1
```

Mature Star

```
uccaucuuguucguucacccucuggaucuuguucgauggcacuccccuuguauccaaucucucucucucucucucucucucucucucucucucauauggugcgaguggaagugccaugaacuugauccagaaggugcucucgaacaaguuacaccuacuuaauaucuacuucuucuuucuccauuacuauuauuaaugaugguuucuuuuuucugauucucauuuuuaauuuauuuauuuguuuuuuguguuug
......................uggaucuuguucgaCggcacu.................... 10 1 JCK-3
......................uggaucuuguucgauggcacuA................... 18 1 LCK-2
......................uggaucuuguucgauggcCcuc................... 3 1 LCK-3
......................uggCucuuguucgauggcacuc................... 3 1 LCK-2
......................uggaucuuguucgCuggcacuc................... 17 1 JCK-3
......................uggaucuuguucgauggcacuc................... 8004 0 LT-3
......................uggaucuuguucgauggcaAuc................... 1 1 JCK-3
......................uggaucuuguucgCuggcacuc................... 17 1 LT-1
......................uggaucuuguucgCuggcacuc................... 16 1 JCK-1
......................uggaucuuguucgauggcaUuc................... 3 1 LT-1
......................uggaucuugCucgauggcacuc................... 1 1 JCK-3
......................uggaucuuUuucgauggcacuc................... 1 1 LT-3
......................uggaucuuguucgauggcacuU................... 147 1 JCK-2
......................uggaucuuguCcgauggcacuc................... 1 1 JCK-3
......................uAgaucuuguucgauggcacuc................... 5 1 LT-1
......................uggaucuuUuucgauggcacuc................... 1 1 JCK-3
......................uggaucuuguucgaugUcacuc................... 2 1 JCK-3
......................uggaucuuguucgauggcCcuc................... 7 1 LT-2
......................uggaucuuguucAauggcacuc................... 8 1 JCK-2
......................uAgaucuuguucgauggcacuc................... 4 1 JCK-1
......................uggaucuuguucgauggcacNc................... 1 1 JCK-1
......................uggGucuuguucgauggcacuc................... 1 1 JCK-2
......................uggCucuuguucgauggcacuc................... 22 1 JCK-3
......................uggaucuuguucgauAgcacuc................... 26 1 JCK-3
......................uggaucuuAuucgauggcacuc................... 2 1 LT-1
......................uggauUuuguucgauggcacuc................... 10 1 JCK-3
......................uggaCcuuguucgauggcacuc................... 3 1 JCK-1
......................uggaucuuguucgauggcacuc................... 785 0 LCK-1
......................uggaucuuguucgauggcacuG................... 8 1 JCK-1
......................uggaucuuguucgauggcacuN................... 6 1 JCK-1
......................uggaucuuguucgauggcacuA................... 17 1 LT-2
......................uggaucuuguAcgauggcacuc................... 1 1 JCK-1
......................uggaucuuguucgauggcacuA................... 58 1 LCK-3
......................uggCucuuguucgauggcacuc................... 30 1 JCK-2
......................uUgaucuuguucgauggcacuc................... 3 1 LT-3
......................uggaucuuguucgauggcacuc................... 3568 0 LCK-3
......................uggaucuuguucgauggcacCc................... 2 1 LT-1
......................uggaucuuUuucgauggcacuc................... 1 1 JCK-2
......................uggaucuuguucAauggcacuc................... 3 1 LT-2
......................uggaucuuguucgCuggcacuc................... 1 1 JT-1
......................uggaucuuguucgauggcaGuc................... 1 1 JCK-1
......................uggaucuuguucgaCggcacuc................... 2 1 JCK-2
......................uggaucCuguucgauggcacuc................... 1 1 LT-1
......................uggaucuuAuucgauggcacuc................... 1 1 JCK-1
......................uggaucuuguucgauggcacCc................... 1 1 JT-1
......................uAgaucuuguucgauggcacuc................... 1 1 LT-2
......................uggCucuuguucgauggcacuc................... 1 1 JT-1
......................uggaCcuuguucgauggcacuc................... 1 1 LCK-1
......................Cggaucuuguucgauggcacuc................... 5 1 JCK-3
......................uggaucuugCucgauggcacuc................... 2 1 LT-2
......................uggaucuuguucgauggUacuc................... 1 1 LT-1
......................uggaucuuguucgauAgcacuc................... 2 1 LT-1
......................uggNucuuguucgauggcacuc................... 6 1 JCK-1
......................Cggaucuuguucgauggcacuc................... 1 1 LT-1
......................uggCucuuguucgauggcacuc................... 29 1 JCK-1
......................uggaucuuguuUgauggcacuc................... 10 1 JCK-2
......................uggaucuuguucgGuggcacuc................... 1 1 LT-2
......................uAgaucuuguucgauggcacuc................... 3 1 JCK-3
......................uAgaucuuguucgauggcacuc................... 3 1 JCK-2
......................uggauUuuguucgauggcacuc................... 10 1 JCK-2
......................uggaucuuguucgauggcNcuc................... 5 1 JCK-1
......................uggaucuuguucgauggcaUuc................... 6 1 LT-2
......................uggCucuuguucgauggcacuc................... 8 1 LCK-3
......................uggaucuuguucAauggcacuc................... 3 1 JCK-1
......................uggaucuuguucgauggUacuc................... 2 1 JCK-1
......................uggaucuuguGcgauggcacuc................... 5 1 JCK-1
......................uggaucuuguucgauAgcacuc................... 42 1 JCK-2
......................uggaucuuguucgauggAacuc................... 1 1 LT-1
......................uggaucuuUuucgauggcacuc................... 2 1 JCK-1
......................uggaucuuguucgauggUacuc................... 1 1 LT-3
```

```
                      Mature                                                                                    Star
uccaucuuguucguucacccucuggaucuuguucgauggcacuccccuuguauccaaucucucucucucucucucucucucucucucauauggugcgaguggaagugccaugaacuugauccagaaggugcucucgaacaaguuacaccuacuuaauaucuacuucuucuuucuccauuacuauuauuaaugaugguuucuuuuuucugauucucauuuuuaauuuauuuauuuguuuuuuguguuug
......................Cggaucuuguucgauggcacuc...............................................................................................................................................................................................................   1   1   LT-2
......................uggaucuuguucgauggcacuc...............................................................................................................................................................................................................   356   0   JT-1
......................uggaucuuguucgauggcacCc...............................................................................................................................................................................................................   4   1   JCK-3
......................uNgaucuuguucgauggcacuc...............................................................................................................................................................................................................   25   1   JCK-2
......................uggaucuuguucgauggcacuU...............................................................................................................................................................................................................   98   1   JCK-3
......................Cggaucuuguucgauggcacuc...............................................................................................................................................................................................................   1   1   JCK-1
......................uggaucuuguucgauggcacuc...............................................................................................................................................................................................................   7340   0   LT-1
......................uggCucuuguucgauggcacuc...............................................................................................................................................................................................................   16   1   LT-1
......................uggaucuuguucgauggcCcuc...............................................................................................................................................................................................................   7   1   JCK-1
......................uggaucuuguuUgauggcacuc...............................................................................................................................................................................................................   3   1   JCK-1
......................ugCaucuuguucgauggcacuc...............................................................................................................................................................................................................   1   1   LT-2
......................uggaucuuguucgauggcaNuc...............................................................................................................................................................................................................   9   1   JCK-1
......................uggaucuuguucgauggcacuA...............................................................................................................................................................................................................   17   1   LCK-1
......................uggaucuuguucgauggcacAc...............................................................................................................................................................................................................   1   1   JCK-1
......................uggaucuuguucgauggcaUuc...............................................................................................................................................................................................................   6   1   LT-3
......................uggaucuuguucgaCggcacuc...............................................................................................................................................................................................................   1   1   LT-1
......................uggaucuuguucgauggcGcuc...............................................................................................................................................................................................................   12   1   JT-1
......................uggGucuuguucgauggcacuc...............................................................................................................................................................................................................   3   1   JCK-1
......................uggaucuuguucgaCggcacuc...............................................................................................................................................................................................................   1   1   JCK-1
......................uggaucuuguucgauggcacuc...............................................................................................................................................................................................................   11364   0   JCK-1
......................uggaucuuguucgauggcCcuc...............................................................................................................................................................................................................   2   1   LCK-1
......................uggaucuugCucgauggcacuc...............................................................................................................................................................................................................   2   1   LT-1
......................uggaucuuguucgaugCcacuc...............................................................................................................................................................................................................   2   1   JCK-3
......................uggaucuuguucgauAgcacuc...............................................................................................................................................................................................................   10   1   JCK-1
......................uggaucuuguucgauggcCcuc...............................................................................................................................................................................................................   6   1   LT-1
......................uggaucCuguucgauggcacuc...............................................................................................................................................................................................................   1   1   JCK-1
......................uggaucuuguucgauggcaUuc...............................................................................................................................................................................................................   33   1   JCK-2
......................uggaucuuguucgaugNcacuc...............................................................................................................................................................................................................   2   1   LT-2
......................uggaucuCguucgauggcacuc...............................................................................................................................................................................................................   1   1   JCK-1
......................uggaucuuguucgauggcacuc...............................................................................................................................................................................................................   177   0   JT-3
......................uggaucuuguucgauggcacCc...............................................................................................................................................................................................................   3   1   JCK-1
......................uggaucuuguucgauggcCcuc...............................................................................................................................................................................................................   24   1   JCK-2
......................uggaucuuguucgauggcacGc...............................................................................................................................................................................................................   1   1   JCK-3
......................uggaucuuguucgCuggcacuc...............................................................................................................................................................................................................   1   1   LCK-2
......................uggaucuuguucgauggcacAc...............................................................................................................................................................................................................   1   1   JCK-3
......................uAgaucuuguucgauggcacuc...............................................................................................................................................................................................................   4   1   LT-3
......................uggaucuuguucUauggcacuc...............................................................................................................................................................................................................   3   1   LT-1
......................ugNaucuuguucgauggcacuc...............................................................................................................................................................................................................   10   1   LT-2
......................uggaucuuguucgauggcacAc...............................................................................................................................................................................................................   1   1   LT-3
......................ugAaucuuguucgauggcacuc...............................................................................................................................................................................................................   3   1   JCK-1
......................uggaucuuguucgauggcacuG...............................................................................................................................................................................................................   4   1   LT-2
......................uggaucuuguucgaugAcacuc...............................................................................................................................................................................................................   2   1   LT-3
......................uggaucuuguucgauggcacuU...............................................................................................................................................................................................................   113   1   LCK-3
......................uggGucuuguucgauggcacuc...............................................................................................................................................................................................................   2   1   JCK-3
......................uggaucuuguucgauggNacuc...............................................................................................................................................................................................................   2   1   JCK-3
......................uggaucuuguucgauggcacuA...............................................................................................................................................................................................................   37   1   JCK-1
......................uggaucuuNuucgauggcacuc...............................................................................................................................................................................................................   2   1   JCK-3
......................uggaucuuguucgCuggcacuc...............................................................................................................................................................................................................   10   1   LT-2
......................uggaCcuuguucgauggcacuc...............................................................................................................................................................................................................   6   1   JCK-2
......................uggaucuuguucgaugAcacuc...............................................................................................................................................................................................................   2   1   JCK-2
......................uggaucuuguucgauggcacAc...............................................................................................................................................................................................................   1   1   JCK-2
......................uggCucuuguucgauggcacuc...............................................................................................................................................................................................................   34   1   LT-2
......................uggaucuuguuNgauggcacuc...............................................................................................................................................................................................................   18   1   JCK-1
......................uggaucuuguucgauggcacuc...............................................................................................................................................................................................................   3507   0   LT-2
......................ugAaucuuguucgauggcacuc...............................................................................................................................................................................................................   1   1   LCK-1
......................uggaucuuguucgauggcacuc...............................................................................................................................................................................................................   14328   0   JCK-3
......................ugAaucuuguucgauggcacuc...............................................................................................................................................................................................................   2   1   LT-1
......................ugAaucuuguucgauggcacuc...............................................................................................................................................................................................................   3   1   JCK-2
......................uggaCcuuguucgauggcacuc...............................................................................................................................................................................................................   1   1   JCK-3
......................uggaucuuguucgaCggcacuc...............................................................................................................................................................................................................   2   1   LT-2
......................uggaucuuguucgauggcacuA...............................................................................................................................................................................................................   72   1   JCK-2
......................uggaucuuguucgauggcaUuc...............................................................................................................................................................................................................   4   1   LCK-3
......................uggaucuuguucgauggcaUuc...............................................................................................................................................................................................................   19   1   JCK-1
......................uggauAuuguucgauggcacuc...............................................................................................................................................................................................................   1   1   JCK-3
......................uggaucuuAuucgauggcacuc...............................................................................................................................................................................................................   1   1   JCK-3
......................uggaucuuguucAauggcacuc...............................................................................................................................................................................................................   4   1   JCK-3
......................ugAaucuuguucgauggcacuc...............................................................................................................................................................................................................   1   1   LCK-3
......................uggaucuuguucgauggcacuU...............................................................................................................................................................................................................   82   1   JCK-1
......................uggaucuuguucgaCggcacuc...............................................................................................................................................................................................................   4   1   JCK-3
......................uggaucuuguucAauggcacuc...............................................................................................................................................................................................................   3   1   LT-3
```

```
                      Mature                                                                              Star
uccaucuuguucguucacccucuggaucuuguucgauggcacuccccuuguauccaaucucucucucucucucucucucucucucucucauauggugcgaguggaagugccaugaacuugauccagaaggugcucucgaacaaguuacaccuacuuaauaucuacuucuucuuucuccauuacuauuauuaaugaugguuucuuuuuucugauucucauuuuuaauuuauuuauuuguuuuuuguguuug
......................uggaucCuguucgauggcacuc............................................................................................................................................................................................................. 5     1  LCK-3
......................uggaucuuguuGgauggcacuc............................................................................................................................................................................................................. 2     1  JCK-1
......................uggauAuuguucgauggcacuc............................................................................................................................................................................................................. 1     1  JCK-2
......................uggaucuuguucgauggcCcuc............................................................................................................................................................................................................. 11    1  LT-3
......................uggaucuuguuUgauggcacuc............................................................................................................................................................................................................. 7     1  JCK-3
......................uggaucuuguucUauggcacuc............................................................................................................................................................................................................. 5     1  JCK-2
......................uggaucuuguucgauggcacuU............................................................................................................................................................................................................. 19    1  LCK-1
......................uUgaucuuguucgauggcacuc............................................................................................................................................................................................................. 3     1  JCK-2
......................uggaucuuguucgauggUacuc............................................................................................................................................................................................................. 2     1  LT-2
......................uUgaucuuguucgauggcacuc............................................................................................................................................................................................................. 3     1  LT-2
......................uggaucuuguucgaugAcacuc............................................................................................................................................................................................................. 2     1  JCK-3
......................uggaucuuguucgauggcacuU............................................................................................................................................................................................................. 121   1  LT-3
......................uggaucuuguucgauggcCcuc............................................................................................................................................................................................................. 11    1  JCK-3
......................uggaucuCguucgauggcacuc............................................................................................................................................................................................................. 1     1  JCK-3
......................uggaucuuguucAauggcacuc............................................................................................................................................................................................................. 1     1  LT-1
......................uggaucuuguucUauggcacuc............................................................................................................................................................................................................. 1     1  JCK-3
......................uggaucuuguucgauggcacuU............................................................................................................................................................................................................. 147   1  LT-1
......................uggaucuuguuUgauggcacuc............................................................................................................................................................................................................. 2     1  LT-1
......................uggaucuuAuucgauggcacuc............................................................................................................................................................................................................. 1     1  LCK-1
......................uggaucuuguucgaugUcacuc............................................................................................................................................................................................................. 1     1  JCK-2
......................uggCucuuguucgauggcacuc............................................................................................................................................................................................................. 1     1  LCK-1
......................uggaucuuguuUgauggcacuc............................................................................................................................................................................................................. 1     1  LT-2
......................uggaucGuguucgauggcacuc............................................................................................................................................................................................................. 1     1  LT-1
......................uggaucCuguucgauggcacuc............................................................................................................................................................................................................. 5     1  JCK-2
......................uggaucuuguucgauAgcacuc............................................................................................................................................................................................................. 1     1  LT-3
......................uggaucuuguucgauggcacCc............................................................................................................................................................................................................. 2     1  JCK-2
......................uggaucuuguucgauggcacuA............................................................................................................................................................................................................. 38    1  LT-1
......................uggaucuuguucgauggcacGc............................................................................................................................................................................................................. 2     1  JCK-2
......................uggaucuuguucgNuggcacuc............................................................................................................................................................................................................. 2     1  LCK-1
......................uggaucuuguucUauggcacuc............................................................................................................................................................................................................. 2     1  LT-3
......................uggaucuuguucgauUgcacuc............................................................................................................................................................................................................. 3     1  JCK-3
......................uggaucuuguucgaugCcacuc............................................................................................................................................................................................................. 1     1  JCK-1
......................uggaucuuguucgauCgcacuc............................................................................................................................................................................................................. 1     1  JCK-2
......................uggauUuuguucgauggcacuc............................................................................................................................................................................................................. 5     1  JCK-1
......................uggaucuugCucgauggcacuc............................................................................................................................................................................................................. 2     1  JCK-2
......................uggaucuuguucgaNggcacuc............................................................................................................................................................................................................. 1     1  LCK-1
......................uggGucuuguucgauggcacuc............................................................................................................................................................................................................. 1     1  LT-2
......................uggauAuuguucgauggcacuc............................................................................................................................................................................................................. 1     1  LT-1
......................uggaucuuguucgaugAcacuc............................................................................................................................................................................................................. 6     1  JCK-1
......................uggaucuuguucgauggcacCc............................................................................................................................................................................................................. 2     1  LT-3
......................Cggaucuuguucgauggcacuc............................................................................................................................................................................................................. 2     1  JCK-2
......................uggaucNuguucgauggcacuc............................................................................................................................................................................................................. 1     1  JCK-3
......................uggaucuuguucgCuggcacuc............................................................................................................................................................................................................. 2     1  LCK-1
......................uggaucuugGucgauggcacuc............................................................................................................................................................................................................. 1     1  LT-1
......................Gggaucuuguucgauggcacuc............................................................................................................................................................................................................. 1     1  JCK-2
......................Nggaucuuguucgauggcacuc............................................................................................................................................................................................................. 1     1  JCK-3
......................uggaCcuuguucgauggcacuc............................................................................................................................................................................................................. 1     1  LT-3
......................uggaucuuguucgauggcacuU............................................................................................................................................................................................................. 72    1  LT-2
......................ugAaucuuguucgauggcacuc............................................................................................................................................................................................................. 2     1  LT-2
......................uAgaucuuguucgauggcacuc............................................................................................................................................................................................................. 1     1  LCK-3
......................uggaucuuguucgauggcacuA............................................................................................................................................................................................................. 44    1  LT-3
......................uggaucuuguucgauggUacuc............................................................................................................................................................................................................. 4     1  JCK-2
......................uggCucuuguucgauggcacuc............................................................................................................................................................................................................. 29    1  LT-3
......................uggaucuuguucgauggcaUuc............................................................................................................................................................................................................. 21    1  JCK-3
......................uggaucuuguucgCuggcacuc............................................................................................................................................................................................................. 4     1  LCK-3
......................uggaucuuguucgauggcacuA............................................................................................................................................................................................................. 53    1  JCK-3
......................ugAaucuuguucgauggcacuc............................................................................................................................................................................................................. 1     1  LT-3
......................uggaucuuguucgauggcacuU............................................................................................................................................................................................................. 30    1  LCK-2
......................uggaucuuguucgauggcGcuc............................................................................................................................................................................................................. 3     1  LCK-3
......................uggaucuuguuAgauggcacuc............................................................................................................................................................................................................. 1     1  LT-3
......................uggaucuuguucgauggcaAuc............................................................................................................................................................................................................. 1     1  LT-3
......................uggaucuuguucgauggcacuc............................................................................................................................................................................................................. 825   0  LCK-2
......................uggaucuuguucgCuggcacuc............................................................................................................................................................................................................. 27    1  LT-3
......................ugAaucuuguucgauggcacuc............................................................................................................................................................................................................. 4     1  JCK-3
......................uggaucuuguucgCuggcacuc............................................................................................................................................................................................................. 33    1  JCK-2
......................uggaucuuguucgauggcacuc............................................................................................................................................................................................................. 143   0  JT-2
......................uNgaucuuguucgauggcacuc............................................................................................................................................................................................................. 15    1  JCK-1
......................uggaucuuguCcgauggcacuc............................................................................................................................................................................................................. 2     1  JCK-1
......................uggaucuuguucgauggcacuc............................................................................................................................................................................................................. 19469 0  JCK-2
......................Cggaucuuguucgauggcacuc............................................................................................................................................................................................................. 1     1  LT-3
```

Mature **Star**

uccaucuuguucguucacccucuggaucuuguucgauggcacuccccuuguauccaaucucucucucucucucucucucucucucucauauggugcgaguggaagugccaugaacuugauccagaaggugcucucgaacaaguuacaccuacuuaauaucuacuucuucuuucuccauuacuauuauuaaugaugguuucuuuuuucugauucucauuuuuaauuuauuuauuuguuuuuuguguuug

......................uggaucuuguucgauggcacuN	1	1	LT-2
......................uggaucuuAuucgauggcacuc	4	1	JCK-2
......................uggaucuuguuNgauggcacuc	1	1	JT-1
......................uNgaucuuguucgauggcacuc	7	1	JCK-3
......................uggaucuuguucgauggGacuc	4	1	JCK-1
......................uggaucuuguucgauggUacuc	6	1	JCK-3
......................uggaucuuguucgauggcacucU	125	1	JCK-2
......................uggaucuuguucgauggcacucU	89	1	JCK-3
......................uggaucuuguucgauggcacucA	28	1	LT-2
......................uggaucuuguucgauggcacuAc	1	1	LT-1
......................uggaucuuguucgauggcacuUc	1	1	JCK-1
......................uggaucuuguucgauggcacucA	6	1	JT-3
......................uggaucuuguucgauggcacucA	70	1	JCK-2
......................uggaucuuguucgauggcacucG	1	1	LT-1
......................uggaucuuguucgauggcacucc	7	0	LCK-3
......................uggaucuuguucgauggcacuUc	2	1	LT-2
......................uggaucuuguucgauggcacucG	3	1	JCK-2
......................uggaucuuguucgauggcacuUc	2	1	LT-3
......................uggaucuuguucgauggcacucU	67	1	LT-2
......................uggaucuuguucgauggcacuAc	1	1	LT-2
......................uggaucuuguucgauggcacucA	55	1	JCK-3
......................uggaucuuguucgauggcacucA	41	1	JCK-1
......................uggaucuuguucgauggcacucU	49	1	LT-3
......................uggaucuuguucgauggcacucc	12	0	LT-2
......................uggaucuuguucgauggcacucc	28	0	LT-1
......................uggaucuuguucgauggcacucA	4	1	JT-2
......................uggaucuuguucgauggcacucU	6	1	JT-3
......................uggaucuuguucgauggcacucA	33	1	LT-3
......................uggaucuuguucgauggcacucA	23	1	LCK-3
......................uggaucuuguucgauggcacucU	88	1	LCK-3
......................uggaucuuguucgauggcacucc	18	0	JCK-3
......................uggaucuuguucgauggcacucU	59	1	LT-1
......................uggaucuuguucgauggcacucG	1	1	LT-3
......................uggaucuuguucgauggcacucG	1	1	LCK-3
......................uggaucuuguucgauggcacucc	21	0	LT-3
......................uggaucuuguucgauggcacucc	34	0	JCK-2
......................uggaucuuguucgauggcacucU	72	1	JCK-1
......................uggaucuuguucgauggcacucA	56	1	LT-1
......................uggaucuuguucgauggcacucc	16	0	JCK-1
......................uggaucuuguucgauggcacucG	1	1	JCK-3
......................uggaucuuguucgauggcacucU	10	1	JT-1
......................uggaucuuguucgauggcacucU	12	1	JT-2
......................uUgaucuuguucgauggcacucc	1	1	JCK-3
......................uggaucuuguucgauggcacuccU	5	1	LCK-3
......................uggaucuuguucgauggcacucAc	4	1	LT-1
......................uggaucuuguucgauggcacuccU	3	1	LT-3
......................uggaucuuguucgauggcacucGc	3	1	LT-1
......................uggaucuuguucgauggcacuccc	4	0	LCK-3
......................uggaucuuguucgauggcacucGc	2	1	LT-2
......................uggaucuuguucgauggcacucAc	7	1	LCK-3
......................uggaucuuguucgauggcacuccU	4	1	LT-1
......................uggaucuuguucgauggcacucUc	1	1	LT-2
......................uggaucuuguucgauggcacuccc	2	0	LT-1
......................uggaucuuguucgauggcacuccU	10	1	LT-2
......................uggaucuuguucgauggcacucAc	10	1	LT-2
......................uggaucuuguucgauggcacucUcc	4	1	LCK-3
......................uggaucuuguucgauggcacuccAc	2	1	JCK-2
......................uggaucuuguucgauggcacuccAc	5	1	LCK-3
......................uggaucuuguucgauggcacuccAc	1	1	JCK-3
......................uggaucuuguucgauggcacucccU	1	1	LT-2
......................uggaucuuguucgauggcacuccAc	1	1	LT-1
......................uggaucuuguucgauggcacuccAc	2	1	JCK-1
......................uggaucuuguucgauggcacucccc	1	0	LT-3
......................uggaucuuguucgauggcacuccAc	4	1	LT-2
......................uggaucuuguucgauggcacucccU	1	1	LT-1
......................uggaucuuguucgauggcacucUccu	1	1	LT-1
.......................ggaucuuguucgauggca	5	0	JT-1
.......................ggaucuuguucgauggcacu	3	0	LCK-3
.......................ggaucuuguucgauggcacu	4	0	JCK-2
.......................ggaucuuguucgauggcacu	10	0	LT-3

Mature Star

```
uccaucuuguucguucacccucuggaucuuguucgauggcacuccccuuguauccaaucucucucucucucucucucucucucucucucauauggugcgaguggaagugccaugaacuugauccagaaggugcucucgaacaaguuacaccuacuuaauaucuacuucuucuuucuccauuacuauuauuaaugaugguuucuuuuuucugauucucauuuuuaauuuauuuauuuguuuuuuguguuug
.......................ggaucuuguucgauggcacu............................................................................................................................................................................................................... 1 0 JCK-1
.......................ggaucuuguucgauggcacu............................................................................................................................................................................................................... 6 0 LT-2
.......................ggaucuuguucgauggcacu............................................................................................................................................................................................................... 7 0 LT-1
.......................Ugaucuuguucgauggcacu............................................................................................................................................................................................................... 1 1 JCK-1
.......................ggaucuuguucgauggcacu............................................................................................................................................................................................................... 3 0 JCK-3
.......................ggaucuuguucgauggcacuc.............................................................................................................................................................................................................. 2 0 LT-1
.......................ggaucuuguucgauggcacuc.............................................................................................................................................................................................................. 3 0 JCK-3
.......................ggaucuuguucgauggcacuc.............................................................................................................................................................................................................. 2 0 JCK-2
.......................ggaucuuguucgauggcacuc.............................................................................................................................................................................................................. 3 0 LT-3
.......................ggaucuuguucgauggcacuc.............................................................................................................................................................................................................. 2 0 LCK-3
.......................ggaucuuguucgauggcacuc.............................................................................................................................................................................................................. 6 0 JCK-1
.......................ggaucuuguucgauggcacucc............................................................................................................................................................................................................. 1 0 JCK-3
.......................ggaucuuguucgauggcacucc............................................................................................................................................................................................................. 3 0 JCK-1
.......................ggaucuuguucgauggcacucc............................................................................................................................................................................................................. 1 0 JCK-2
.......................ggaucuuguucgauggcacucc............................................................................................................................................................................................................. 1 0 LT-1
.......................ggaucuuguucgauggcacuccc............................................................................................................................................................................................................ 3 0 JCK-3
.......................ggaucuuguucgauggcacuccc............................................................................................................................................................................................................ 4 0 JCK-1
........................gaucuuguucgauggcacu............................................................................................................................................................................................................... 2 0 JCK-1
........................gaucuuguucgauggcacu............................................................................................................................................................................................................... 1 0 JCK-3
........................gaucuuguucgauggcacu............................................................................................................................................................................................................... 2 0 LT-2
........................gaucuuguucgauggcacuc.............................................................................................................................................................................................................. 1 0 LT-3
........................gaucuuguucgauggcacuc.............................................................................................................................................................................................................. 4 0 LT-2
........................gaucuuguucgauggcacuc.............................................................................................................................................................................................................. 1 0 JCK-2
........................gaucuuguucgauggcacucc............................................................................................................................................................................................................. 1 0 LT-3
........................gaucuuguucgauggcacucc............................................................................................................................................................................................................. 1 0 LT-2
........................gaucuuguucgauggcacucc............................................................................................................................................................................................................. 5 0 JCK-1
........................gaucuuguucgauggcacucc............................................................................................................................................................................................................. 12 0 JCK-2
........................gaucuuguucgauggcacucc............................................................................................................................................................................................................. 8 0 JCK-3
........................gaucuuguucgauggcacuccc............................................................................................................................................................................................................ 6 0 LT-2
........................gaucuuguucgauggcacuccU............................................................................................................................................................................................................ 1 1 JCK-3
........................gaucuuguucgauggcacuccc............................................................................................................................................................................................................ 2 0 LT-1
........................gaucuuguucgauggcacuccc............................................................................................................................................................................................................ 24 0 JCK-1
........................gaucuuguucgauggcacuccc............................................................................................................................................................................................................ 37 0 JCK-3
........................Aaucuuguucgauggcacuccc............................................................................................................................................................................................................ 1 1 JCK-2
........................gaucuuguucgauggcacuccc............................................................................................................................................................................................................ 6 0 LT-3
........................gaucuuguucgauggcacuccc............................................................................................................................................................................................................ 5 0 LCK-3
........................gaucuuguucgauggcacuccc............................................................................................................................................................................................................ 56 0 JCK-2
.........................aucuuguucgauggcacu............................................................................................................................................................................................................... 1 0 LT-2
.........................aucuuguucgauggcacuc.............................................................................................................................................................................................................. 1 0 JCK-1
.........................aucuuguucgauggcacuc.............................................................................................................................................................................................................. 3 0 LT-3
.........................aucuuguucgauggcacuc.............................................................................................................................................................................................................. 1 0 JCK-3
.........................aucuuguucgauggcacucc............................................................................................................................................................................................................. 3 0 LT-3
.........................aucuuguucgauggcacucU............................................................................................................................................................................................................. 2 1 JCK-2
.........................aucuuguucgauggcacucU............................................................................................................................................................................................................. 1 1 JCK-3
.........................aucuuguucgauggcacucc............................................................................................................................................................................................................. 1 0 JCK-2
.........................aucuuguucgauggcacucc............................................................................................................................................................................................................. 1 0 JCK-1
.........................aucuuguucgauggcacucU............................................................................................................................................................................................................. 1 1 LT-1
.........................aucuuguucgauggcacucc............................................................................................................................................................................................................. 1 0 JCK-3
.........................aucuuguucgauggcacucc............................................................................................................................................................................................................. 2 0 LT-2
.........................aucuuguucgauggcacuccc............................................................................................................................................................................................................ 1 0 LT-1
.........................aucuuguucgauggcacuccc............................................................................................................................................................................................................ 5 0 JCK-2
.........................aucuuguucgauggcacuccc............................................................................................................................................................................................................ 2 0 JCK-3
.........................aucCuguucgauggcacuccc............................................................................................................................................................................................................ 1 1 LT-3
.........................aucuuguucgauggcacuccc............................................................................................................................................................................................................ 1 0 LT-3
.........................aucuuguucgauggcacucccc........................................................................................................................................................................................................... 2 0 LT-3
..........................ucuuguucgauggcacuc.............................................................................................................................................................................................................. 1 0 JCK-3
..........................ucuuguucgauggcacucc............................................................................................................................................................................................................. 4 0 LCK-3
..........................ucuuguucgauggcacuccc............................................................................................................................................................................................................ 2 0 LT-3
...................................................aucAaaucucucucucucucucucucuc........................................................................................................................................................................... 1 1 LT-1
....................................................Accaaucucucucucucucuc................................................................................................................................................................................. 1 1 LT-3
........................................................aucucucucucucucuGucuc............................................................................................................................................................................. 1 1 LT-3
.........................................................ucucucucucuNucucucucucucuc....................................................................................................................................................................... 4 1 JCK-1
...........................................................ucucucucucuNucucucucucucuc..................................................................................................................................................................... 4 1 JCK-1
.............................................................ucucucucucuNucucucucucucuc................................................................................................................................................................... 4 1 JCK-1
...............................................................ucucucucucuNucucucucucucuc................................................................................................................................................................. 4 1 JCK-1
....................................................................cucucucucucucucuGucuca................................................................................................................................................................ 1 1 LT-3
.......................................................................ucucucucucGcucucuca................................................................................................................................................................ 1 1 JCK-1
............................................................................................uggugcgaguggaagugccaugaacu.................................................................................................................................... 1 0 LT-3
..............................................................................................gugcgaguggaagugccaugaacuug.................................................................................................................................. 5 0 LCK-3
................................................................................................gcgaguggaagugccaugaacuugau................................................................................................................................ 2 0 LCK-3
```

Mature / **Star**

uccaucuuguucguucacccucuggaucuuguucgauggcacuccccuuguauccaaucucucucucucucucucucucucucucucucauauggugcgaguggaagugccaugaacuugauccagaaggugcucucgaacaaguuacaccuacuuaauaucuacuucuucuuucuccauuacuauuauuaaugaugguuucuuuuuucugauucucauuuuuaauuuauuuauuuguuuuuuguguuug

cgaguggaagugccaugaacuugauccaga	1	0	LT-1
cgaguggaagugccaugaacuugauccagC	1	1	LT-1
gaguggaagugccaugaacuugaucc	1	0	JCK-3
gaguggaagugccaugaacuugaucc	1	0	JCK-2
gaguggaagugccaugaacuugauccaga	2	0	JCK-3
gaguggaagugccaugaacuugauccaga	1	0	JCK-1
gaguggaagugccaugaacuugauccaga	1	0	JCK-2
gaguggaagugccaugaacuugauccagaa	1	0	JCK-1
gaguggaagugccaugaacuugauccagaa	1	0	JCK-3
aguggaagugccaugaacuuga	1	0	LT-3
aguggaagugccaugaacuugauc	1	0	JCK-2
aguggaagugccaugaacuugauc	1	0	JCK-1
aguggaagugccaugaacuugauccaga	2	0	LCK-3
aguggaagugccaugaacuugauccaga	4	0	JCK-2
aguggaagugccaugaacuugauccaga	1	0	JCK-3
guggaagugccaugaacuugaucc	2	0	JT-2
guggaagugccaugaacuugaucc	1	0	JCK-1
guggaagugccaugaacuugaucc	4	0	JT-3
guggaagugccaugaacuugaucc	2	0	JCK-3
guggaagugccaugaacuugauccaU	3	1	LCK-3
guggaagugccaugaacuugauccaga	4	0	JCK-2
guggaagugccaugaacuugauccagU	4	1	JCK-1
guggaagugccaugaacuugauccaga	1	0	JCK-3
guggaagugccaugaacuugauccaga	17	0	LCK-2
guggaagugccaugaacuugauccagU	1	1	LCK-3
guggaagugccaugaacuugauccaga	27	0	LCK-1
guggaagugccaugaacuugauccagU	1	1	JCK-3
guggaagugccaugaacuugauccagU	1	1	JCK-2
guggaagugccaugaacuugauccaga	1	0	LT-2
uggaaUugccaugaacuuga	1	1	JCK-2
uggaaUugccaugaacuuga	1	1	JCK-3
uggaaUugccaugaacuugau	2	1	JCK-2
uggaaUugccaugaacuugau	1	1	JCK-1
uggaagugccaugaacuugau	2	0	LT-3
uggaaUugccaugaacuugau	3	1	JCK-3
uggaagugccaugaacuugauU	1	1	JCK-2
uggaaUugccaugaacuugauc	7	1	JCK-2
uggaaUugccaugaacuugauc	3	1	JCK-1
uggaagugccaugaacuugauc	1	0	LT-2
uggaagugccaugaacuugauU	1	1	JCK-3
uggaagugccaugaacuugauA	1	1	LT-1
uggaagugccaugaacuugauc	1	0	JCK-1
uggaagugccaugaacuugauc	2	0	JCK-2
uggaagugccaugaacuugauc	4	0	JCK-3
uggaagugccaugaacuugauU	2	1	LT-3
uggaagugccaugaacuugaucc	3	0	LT-1
uggaaUugccaugaacuugaucc	4	1	JCK-1
uggaaUugccaugaacuugaucc	21	1	JCK-2
uggaagugccaugaacuugaucc	2	0	LT-2
uggaagugccaugaacuugaucc	3	0	LT-3
uggaaUugccaugaacuugaucc	6	1	JCK-3
uggaaUugccaugaacuugaucc	2	1	JT-3
uggaagugccaugaacuugaucc	3	0	JCK-1
uggaagugccaugaacuugaucc	2	0	JCK-3
uggaagugccaugaacuugaucc	5	0	LCK-3
uggaaUugccaugaacuugaucc	2	1	JT-2
uggaagugccaugaacuugaucc	7	0	JCK-2
uggaaUugccaugaacuugaucca	8	1	JCK-2
uggaaUugccaugaacuugaucca	8	1	JCK-3
uggaaUugccaugaacuugaucca	3	1	JCK-1
uggaagugccaugaacuugauccagU	1	1	LT-3
uggaagugccaugaacuugauccagC	2	1	LT-2
gaagugccaugaacuugauccaA	1	1	LT-1
aaUugccaugaacuugau	3	1	JCK-1
aagugccaugaacuugau	2	0	JCK-1
aagugccaugaacuugau	2	0	JT-3
aaUugccaugaacuugau	2	1	JCK-3
aagugccaugaacuugau	6	0	JCK-2
aaUugccaugaacuugau	3	1	JT-3
aaUugccaugaacuugau	19	1	JT-1

Mature / Star			
uccaucuuguucguucacccuc**uggaucuuguucgauggcacuc**cccuuguauccaaucucucucucucucucucucucucucucucucauauggugcgaguggaa**gugccaugaacuugauccaga**aggugcucucgaacaaguuacaccuacuuaauaucuacuucuucuuucuccauuacuauuauuaaugaugguuucuuuuuucugauucucauuuuuaauuuauuuauuuguuuuuuguguuug			
aaUugccaugaacuugau	9	1	JCK-2
aagugccaugaacuugUu	1	1	LT-1
aagugccaugaacuugau	3	0	LT-2
aagugccaugaacuugau	7	0	LT-1
aagugccaugaacuugau	5	0	LT-3
aagugccaugaacuuUau	4	1	LCK-3
aaUugccaugaacuugau	1	1	JT-2
aagugccaugaacuugUu	2	1	LT-3
aagugccaugaacuugau	5	0	JCK-3
aagugccaugaacuugaA	2	1	LT-3
aagugccaugaacuugauc	1	0	LT-1
aagugccaugCacuugauc	1	1	JCK-1
aagugccaugaacuugauc	3	0	JT-2
aagugccaugaacuugauc	1	0	LT-2
aagugccaugaacuugauc	23	0	JCK-3
aagugccaugaacuugauU	2	1	LT-2
aagugccaugaacuugauc	8	0	JT-3
aagugccaugaacuugauU	6	1	LT-1
aaUugccaugaacuugauc	21	1	JT-2
aagugccaugaacuugauA	1	1	LT-3
aaUugccaugaacuugauc	15	1	JCK-2
aagugccaugaacuugauc	22	0	JCK-1
aagugccaugaacuugauc	15	0	JCK-2
aaUugccaugaacuugauc	12	1	JCK-3
aagugccaugaacuugauc	8	0	LT-3
aagugccaugaacuugauU	4	1	JCK-3
aagugccaugaacuugauU	5	1	LT-3
aaUugccaugaacuugauc	26	1	JT-3
aCgugccaugaacuugauc	1	1	LT-2
aagugccaugaacuugauU	2	1	JCK-1
aagugccaugaacuugauU	1	1	JCK-2
aaUugccaugaacuugauc	28	1	JT-1
aaUugccaugaacuugauc	6	1	JCK-1
aagugccaugaacuugaucU	1	1	JCK-1
aagugccaugaacuugaucU	3	1	LT-2
aagugccaugCacuugaucc	1	1	LT-1
aagugccaugaacuugaucU	3	1	JCK-3
aagugccaugaacuugaucc	93	0	LT-3
aagugccaugGacuugaucc	1	1	LT-3
aagugccaugaacuugaucc	17	0	LCK-1
aagugccaugaacuugaucc	64	0	LT-2
aagugccaugaacuugaucc	108	0	JCK-2
aagugccaugaacuugaucc	21	0	LCK-2
aCgugccaugaacuugaucc	1	1	JCK-3
aagugccaugaacuugaucA	3	1	JCK-3
aaUugccaugaacuugaucc	25	1	JCK-3
aaUugccaugaacuugaucc	41	1	JCK-2
aCgugccaugaacuugaucc	1	1	LT-2
aagugccaugaacuugaucc	51	0	LT-1
aagugccaugaacuugaucU	1	1	LT-3
aaUugccaugaacuugaucc	21	1	JT-3
aNgugccaugaacuugaucc	1	1	JCK-3
aagugcAaugaacuugaucc	1	1	JCK-3
aagugccaugaacuugaucc	105	0	JCK-3
aagugccaugaacuugaucU	3	1	JCK-2
aagugccaugaacuugaucc	62	0	JCK-1
aaUugccaugaacuugaucc	26	1	JCK-1
aagugccaugaacuugaucA	2	1	JCK-1
aagugccaugaacuugaucc	89	0	LCK-3
aaUugccaugaacuugaucc	20	1	JT-2
aagugccaugaacuugaucU	6	1	LCK-3
aagugccaugaCcuugaucc	1	1	JCK-2
Cagugccaugaacuugaucc	1	1	LT-2
aagugcAaugaacuugaucc	1	1	JCK-2
aagugccaugaacuugaucU	2	1	LT-1
aCgugccaugaacuugaucca	1	1	LT-2
aagugcAaugaacuugaucca	1	1	JCK-2
aaUugccaugaacuugaucca	10	1	JCK-1
aagugccaugaacuugaucca	7	0	JT-2
aagugccaugaacuugaucca	5	0	LCK-3

Mature **Star**

uccaucuuguucguucacccucuggaucuuguucgauggcacucccuuguauccaaucucucucucucucucucucucucucucucauauggugcgaguggaagugccaugaacuugauccagaaggugcucucgaacaaguuacaccuacuuaauaucuacuucuucuuucuccauuacuauuauuaaugaugguuucuuuuuucugauucucauuuuuaauuuauuuauuuguuuuuuguguuug

Sequence	Count	Mismatch	Library
aagugccaugaacuugaucca	41	0	LT-3
aaUugccaugaacuugaucca	14	1	JCK-3
aaNugccaugaacuugaucca	1	1	LT-2
aagugccaugaacuugaucca	6	0	JT-3
aagugccaugaacuugaucca	120	0	JCK-3
aagugccaugaacuugauccC	1	1	JCK-2
aagugUcaugaacuugaucca	1	1	JCK-3
aagugccaugaacuugauccU	2	1	JCK-2
aagugccaugaacuugaucca	33	0	LT-1
aaUugccaugaacuugaucca	19	1	JCK-2
aagugccaugaacuugauccU	1	1	LT-1
aagugccaugaacuugaGcca	1	1	JCK-1
aagugccCugaacuugaucca	1	1	JCK-3
aagugccaugaacuugaucca	93	0	JCK-1
aaguAccaugaacuugaucca	1	1	LT-2
aagugccaugaacuugaucca	33	0	LT-2
Cagugccaugaacuugaucca	1	1	JCK-3
aagugccaugaacuugaucca	184	0	JCK-2
aagugccaugaacuugauccC	2	1	LT-3
aagugccaugaacuugauccC	2	1	JCK-3
aNgugccaugaacuugaucca	1	1	JT-2
aagugccaugaacuugauccU	2	1	LT-3
aagugccaugaGcuugaucca	1	1	JCK-1
aagugccaugaacuugauccaA	2	1	JCK-1
aagugccaugaacuugauccag	2	0	LT-2
aagugccaugaacuugauccaU	2	1	JCK-2
aagugccaugaacuugauccag	47	0	JCK-1
aagugccaugaacuugauccaU	6	1	JCK-3
aagugccaugaacuugauccag	88	0	JCK-2
aagugccaugaacuugauccaA	2	1	JCK-2
aagugccaugaacuugauccag	27	0	LT-1
aagugccaugaacuugauccag	71	0	JCK-3
Cagugccaugaacuugauccag	3	1	LT-1
aagugccaugaacuugauccag	29	0	LT-3
aagugccaugaacuugauccag	17	0	LCK-3
aagugccaugaacuugaGccag	1	1	JCK-1
aagugccaugaacuugauccaU	3	1	JCK-1
aagugccaugaacuugauccaA	1	1	JCK-3
aagugccaugaacuugauccaA	1	1	LT-3
aagugccaugaacuugauccaga	5	0	JCK-1
aagugccaugaacuugauccagC	2	1	JCK-1
aagugccaugaacuugauccagC	1	1	LT-1
aagugccaugaacuugauccagC	1	1	JCK-3
aagugccaugaacuugauccagU	1	1	JCK-3
aagugccaugaacuugauccagU	1	1	JCK-2
aagugccaugaacuugauccaCa	3	1	JCK-3
aagugccaugaacuugauccagU	2	1	LT-1
aagugccaugaacuugauccagC	1	1	JCK-2
aagugccaugaacuugauccagU	2	1	JCK-1
aagugccaugaacuugauccaga	4	0	JCK-3
aagugccaugaacuugauccaga	2	0	LT-1
aagugccaugaacuugauccaCa	2	1	JCK-2
aagugccaugaacuugauccaga	11	0	JCK-2
aagugccaugaacuugauccaga	2	0	LT-3
aagugccaugaacuugauccaCa	1	1	JCK-1
aagugccaugaacuugauccaAaaggug	1	1	JCK-1
aagugccaugaacuugauccagaaggug	1	0	JCK-3
.agugccaugaacuugauc	14	0	LT-3
.agugccaugaacuugauc	15	0	LT-2
.agugccaugaacuugauU	2	1	LT-3
.agugccCugaacuugauc	1	1	LT-2
.agugccaugaacuugauU	1	1	JCK-3
.agugccaugaacCugauc	1	1	JCK-2
.agugccaugaacuugauc	5	0	JCK-1
.agugccaugaacuugauc	19	0	LCK-2
.agugccaugaacuugauA	1	1	LT-1
.agugccaugaacuugauc	5	0	JCK-3
.agugccaugaacuugauc	6	0	LCK-3
.agugccaugaacuugauc	10	0	JCK-2
.agugccaugaacuugauc	11	0	LCK-1

Mature **Star**

uccaucuuguucguucacccucuggaucuuguucgauggcacuccccuuguauccaaucucucucucucucucucucucucucucucucauauggugcgaguggaagugccaugaacuugauccagaaggugcucucgaacaaguuacaccuacuuaauaucuacuucuucuuucuccauuacuauuauuaaugaugguuucuuuuuucugauucucauuuuaauuuauuuauuuguuuuuuguguuug			
agugccaugaacuNgauc	1	1	LCK-1
agugccaugaacuugauc	7	0	LT-1
aUugccaugaacuugaucc	2	1	JCK-3
agugccaugaacuugaucc	19	0	LT-1
agugccaugaacuugaucc	51	0	LT-2
aUugccaugaacuugaucc	3	1	JCK-2
agugccaugaacuugaucA	2	1	LT-2
agugccaugaacuugaucc	30	0	JCK-2
agugccaugaacuugaucc	3	0	JT-2
agugccaugaacuugaucc	22	0	JCK-3
agugccaugaacuugaucc	41	0	LCK-1
agugccaugaacuugaucc	47	0	LCK-2
agugccaugaacuugaucc	6	0	JT-3
agugccaugaacuugaucU	1	1	LT-1
agugccaugaacuugaucc	45	0	LCK-3
agugccaugaacuugaucc	27	0	JCK-1
agugccaugaacuugGucc	1	1	LT-2
agugccaugaacuugaucc	46	0	LT-3
agugccaugaacuugCucca	1	1	LT-2
agugccaugaacuugaucca	25	0	JCK-2
aUugccaugaacuugaucca	1	1	JCK-3
agugccaugaacuugaucca	18	0	LT-3
agugccaugaacuugaucca	27	0	LT-2
agugccaugaacuugaucca	10	0	JCK-1
agugccaugaacuugaucca	11	0	JCK-3
agugccaugaacuugaucca	51	0	LCK-3
agugccaugaacuugaucca	20	0	LT-1
agugccaugaacuugaucca	20	0	LCK-1
aUugccaugaacuugaucca	1	1	JCK-1
aUugccaugaacuugaucca	2	1	JCK-2
agugccaugaacuugaucca	5	0	JT-2
agugccaugaacuugaucca	22	0	LCK-2
agugccaugaacuugaucca	12	0	JT-3
Cgugccaugaacuugaucca	1	1	LCK-3
aguAccaugaacuugaucca	1	1	JCK-2
agugccCugaacuugauccag	1	1	JCK-2
agugccaugaacuugauccaU	4	1	JCK-1
agugccaugaacuugaCccag	1	1	LT-3
aUugccaugaacuugauccag	2	1	JCK-3
agugccaugaacuugauccag	179	0	JCK-2
agugccaugaacuugauccaA	1	1	LT-1
agugccaugaacuugauccag	23	0	LCK-3
agugccaugaacuugauccag	58	0	LT-3
agugccaugaacuugauccag	120	0	JCK-3
agugccaugaacuugauccaA	1	1	JCK-3
agugccaugaacuugauccaU	3	1	JCK-2
agugccaugaacuugauccaA	1	1	JCK-1
agugccaugaacuugauccag	28	0	LT-2
aUugccaugaacuugauccag	1	1	JCK-2
agugccaugaacuugauccaU	4	1	JCK-3
Cgugccaugaacuugauccag	1	1	JCK-3
agugccGugaacuugauccag	1	1	JCK-3
aguAccaugaacuugauccag	1	1	LT-3
agugccaugaacuugauccCg	1	1	JCK-2
agugccaugaacuugauccag	50	0	LT-1
agugccaugaacuugauccaU	2	1	LT-1
agugccaugaacuugauccaA	3	1	JCK-2
agugccaugaacuugauccag	115	0	JCK-1
agugccaugaacuugauccaga	9	0	JCK-1
agugccaugaacuugauccagU	6	1	JCK-1
agugccaugaacuugauccaga	2	0	LT-2
agugccaugaacuugauccagU	2	1	JCK-3
agugccaugaacuugauccagU	4	1	JCK-2
agugccaugaacuugauccaga	2	0	LT-1
agugccaugaacuugauccaAa	1	1	JCK-2
agugccaugaacuugauccaga	5	0	JCK-3
agugccaugaacuugauccagC	1	1	JCK-2
agugccaugaacuugauccaga	6	0	LT-3
Cgugccaugaacuugauccaga	2	1	JCK-3
agugccaugaacuugauccaga	16	0	JCK-2

Mature **Star**

uccaucuuguucguucacccucuggaucuuguucgauggcacuccccuuguauccaaucucucucucucucucucucucucucucucucucauauggugcgaguggaagugccaugaacuugauccagaaggugcucucgaacaaguuacaccuacuuaauaucuacuucuucuuucuccauuacuauuauuaaugaugguuucuuuuuucugauucucauuuuuaauuuauuuauuuguuuuuuguguuug

Cgugccaugaacuugauccaga	1	1	LT-1
agugccaugaacuugauccaAa	1	1	JCK-3
Cgugccaugaacuugauccaga	4	1	JCK-1
Cgugccaugaacuugauccaga	4	1	JCK-2
Ugugccaugaacuugauccaga	1	1	LT-3
agugccaugaacuugauccagUa	1	1	LT-1
Uugccaugaacuugaucc	32	1	JCK-2
gugccaugaacuugaucc	34	0	LT-1
gugccaugaacuugaucc	40	0	JCK-3
gugccaugaacuugaucU	1	1	LT-3
gugccaugaacuugaucc	51	0	JCK-2
gugccaugaacuugaucc	41	0	LCK-3
gugccaugaacuugaucA	8	1	JCK-2
Augccaugaacuugaucc	2	1	LCK-1
gugccaugaacuugaucc	34	0	LCK-1
Augccaugaacuugaucc	1	1	LT-2
gugccaugaacuugaucc	35	0	LT-2
Uugccaugaacuugaucc	19	1	JCK-3
gugccaugaacuugaucc	40	0	LCK-2
gugccaugaacuugaucc	11	0	JT-1
gugccaugaacuugaucA	5	1	JCK-3
Uugccaugaacuugaucc	15	1	JCK-1
gugccaugaacuugaucc	69	0	LT-3
gugccaugaacuugaucc	26	0	JCK-1
Uugccaugaacuugaucc	3	1	JT-1
Uugccaugaacuugaucca	2	1	JCK-3
gugccaugaacuugaucca	6	0	JCK-3
gugccaugaacuugaucca	22	0	LT-3
gugccaugaacuugaucca	27	0	LT-2
Uugccaugaacuugaucca	3	1	JCK-2
gugccaugaacuugaucca	11	0	JCK-2
gugccaugaacuugaucca	14	0	JT-1
gugccaugaacuugaucca	6	0	JCK-1
gugccaugaacuugaucca	19	0	LCK-3
gugccaugaacuugaucca	14	0	LT-1
Uugccaugaacuugaucca	5	1	JCK-1
gugccaugaCcuugauccag	1	1	JT-3
gugccaugaacuugauccaA	3	1	LT-1
gugccaugaacuugauccaU	1	1	JCK-2
gugccaugaacuugauccag	46	0	LCK-1
gugccaugCacuugauccag	1	1	LT-1
gugccaugaacuugauccaA	7	1	JT-3
gugccaugaacuugauccaA	4	1	JT-2
gugccaugaacuugauccag	125	0	LT-3
Uugccaugaacuugauccag	66	1	JCK-3
Uugccaugaacuugauccag	79	1	JCK-2
Uugccaugaacuugauccag	6	1	JT-2
Uugccaugaacuugauccag	34	1	JCK-1
gugccaugaacuugauccag	221	0	JCK-2
gugccaugaCcuugauccag	1	1	LT-2
gugccaugaacuugauccaA	5	1	LT-3
gugccaugaacuugauccaA	8	1	JCK-2
gugccaugaacuugauccaU	1	1	JCK-1
gugccaugaCcuugauccag	1	1	JCK-2
gugccaugaacuugauccag	63	0	LCK-3
Uugccaugaacuugauccag	1	1	LT-2
gugccaugaacuugauccaA	3	1	JCK-1
Uugccaugaacuugauccag	1	1	LT-3
gugccaugaacuugauccag	56	0	LCK-2
Augccaugaacuugauccag	1	1	JCK-2
Augccaugaacuugauccag	1	1	JT-3
gugccaugaacuugauccag	47	0	JT-1
Uugccaugaacuugauccag	10	1	JT-3
gugccaugaacuugauccag	163	0	JCK-3
gugccaugaacuugauccag	103	0	LT-1
Uugccaugaacuugauccag	20	1	JT-1
gugccaugaacuugauccaU	6	1	LT-2
gugccaugaaAuugauccag	1	1	LT-2
gugccaugaacuugauccag	16	0	JT-2
Augccaugaacuugauccag	1	1	LCK-2

Mature **Star**

uccaucuuguucguucacccucuggaucuuguucgauggcacuccccuuguauccaaucucucucucucucucucucucucucucucauauggugcgaguggaagugccaugaacuugauccagaaggugcucucgaacaaguuacaccuacuuaauaucuacuucuucuuucuccauuacuauuauuaaugaugguuucuuuuuucugauucucauuuuuaauuuauuuauuuguuuuuuguguuug

gugccaugaacuugauccag	26	0	JT-3
gugccaugaacuugauccag	138	0	JCK-1
gugccaugaacuugauccag	84	0	LT-2
gugccaugaacCugauccag	1	1	LT-2
gugccaugaacuugauccaU	2	1	JCK-3
gugccaugaacuugCuccag	1	1	LT-3
gugccaugaacuugauccaA	4	1	JCK-3
gugccaugaacuugauccaUa	2	1	LT-1
gugccaugaacuugauccaAa	7	1	LT-3
gugccaugaacuugauccCga	1	1	JT-1
guAccaugaacuugauccaga	1	1	JCK-3
gugccaugaacuugauccagC	1	1	LT-2
gugccaugaacCugauccaga	1	1	JCK-1
gugccaugaacuugauccaUa	1	1	JCK-2
Augccaugaacuugauccaga	3	1	JCK-1
gugccaugaacuugauccagU	6	1	JCK-1
gugcUaugaacuugauccaga	2	1	JCK-2
gugccaugaacuugauccaAa	1	1	LCK-3
gugccaugaacuugauccaga	463	0	LT-2
gugccaugaacuugauccCga	1	1	LCK-1
gugccaugaacuugauccCga	1	1	LCK-3
Uugccaugaacuugauccaga	1141	1	JCK-2
Uugccaugaacuugauccaga	871	1	JCK-3
gugcUaugaacuugauccaga	3	1	JCK-3
gugccaugNacuugauccaga	1	1	JCK-3
gugccaugaacuuUauccaga	1	1	JCK-1
gugccaugaacuugauccaga	444	0	LT-1
gugccaugaacuugauccagU	8	1	JCK-2
gugccaugCacuugauccaga	1	1	JCK-3
gugccCugaacuugauccaga	4	1	JCK-2
gugccaugaacNugauccaga	1	1	JT-1
Augccaugaacuugauccaga	7	1	JCK-3
gNgccaugaacuugauccaga	1	1	JCK-3
gugccaugaacuugauccaUa	1	1	LT-3
gugccaugaCcuugauccaga	1	1	LCK-3
gugccaugaacuugauccaga	295	0	LCK-3
Uugccaugaacuugauccaga	6	1	JT-3
Augccaugaacuugauccaga	3	1	LT-3
Augccaugaacuugauccaga	1	1	LT-1
gugccaugaacuugauccaga	107	0	LCK-2
gugccaugaacuugauccaga	56	0	JT-2
Cugccaugaacuugauccaga	1	1	LT-3
gugccaugaacuugauccCga	2	1	JCK-1
gugccaugaacuugCuccaga	2	1	LT-3
gugccaugaacuugauccagC	7	1	LT-3
gugccaugaacuugaucGaga	2	1	JCK-1
Uugccaugaacuugauccaga	666	1	JCK-1
gugccaugaacuCgauccaga	1	1	JCK-1
gugcAaugaacuugauccaga	1	1	JT-1
gugccaugaacuugauccCga	1	1	LT-1
gugccCugaacuugauccaga	1	1	LT-3
gugccaugaacuugauccagG	1	1	JCK-2
gNgccaugaacuugauccaga	2	1	JCK-1
gugccaugaacuugauccaga	90	0	LCK-1
gugccaugaacuugauccaAa	4	1	JCK-2
gugccaugaacuugauccagU	9	1	JT-1
gugccaugGacuugauccaga	1	1	JCK-1
gugccaugaacuugauccaAa	5	1	LT-1
gugccaugaacuugauccaga	2129	0	JCK-3
gugccNugaacuugauccaga	1	1	LCK-1
gugccaugaacNugauccaga	7	1	JCK-1
gugccaugaacuugauccagU	25	1	LCK-2
gugccaugaacuugCuccaga	2	1	JCK-2
gugccaugaacuugauccaga	649	0	LT-3
Uugccaugaacuugauccaga	1	1	JT-1
gugccaugaacuugauccaAa	9	1	JCK-3
guAccaugaacuugauccaga	2	1	JCK-2
gugccaugaacuugauccagU	22	1	LT-3
gugccCugaacuugauccaga	1	1	JT-1
gugccaugaacuugauccagU	5	1	LT-1

Mature **Star**

uccaucuuguucguucacccucuggaucuuguucgauggcacuccccuuguauccaaucucucucucucucucucucucucucucucucauauggugcgaguggaagugccaugaacuugauccagaaggugcucucgaacaaguuacaccuacuuaauaucuacuucuucuuucuccauuacuauuauuaaugaugguuucuuuuuucugauucucauuuuuaauuuauuuauuuguuuuuuguguuug

gCgccaugaacuugauccaga	1	1	LT-3
gugccaugaacuugCuccaga	1	1	LT-1
gugccaugaacuugauccaAa	4	1	JCK-1
gugccaugaacuugauccNga	1	1	JCK-1
gugccaugaacuugauccagU	9	1	LT-2
gCgccaugaacuugauccaga	2	1	JCK-3
Cugccaugaacuugauccaga	4	1	JCK-2
gNgccaugaacuugauccaga	3	1	JCK-2
gugccaugaacuugauccaga	134	0	JT-1
gugccaugaacuugauccaNa	3	1	JCK-1
gugUcaugaacuugauccaga	2	1	JCK-2
gugccaugaacuugauccaga	43	0	JT-3
gugccaugaCcuugauccaga	2	1	JCK-1
gugccaugaCcuugauccaga	2	1	JCK-2
guNccaugaacuugauccaga	1	1	LT-2
gugccaugaacuugaucUaga	2	1	JCK-1
gugccaugaacuugauccagC	1	1	JCK-2
Uugccaugaacuugauccaga	4	1	JT-2
gugccaugaacuugauccaAa	2	1	LT-2
gugccaugaacuugauccagU	16	1	LCK-1
gugccaugaacuCgauccaga	1	1	JCK-3
gugccaugaacuugauccaCa	1	1	JCK-1
Augccaugaacuugauccaga	4	1	JCK-2
gugccaugaacuugaucGaga	1	1	JCK-3
Cugccaugaacuugauccaga	4	1	JCK-3
Cugccaugaacuugauccaga	1	1	JCK-1
gugccaugaacuugauccagU	10	1	JCK-3
gugccCugaacuugauccaga	2	1	LT-2
Uugccaugaacuugauccaga	1	1	LCK-3
gugccaugaacuCgauccaga	1	1	JCK-2
gugccaugaacuugauccaga	1669	0	JCK-1
gugcAaugaacuugauccaga	2	1	JCK-1
gugccaugaacuugauccagC	2	1	LT-1
gugccaugaacuugauccaga	2906	0	JCK-2
gugccaugaacuugCuccaga	1	1	LT-2
gugccCugaacuugauccaga	2	1	LT-1
gugccaugaacuugauccGga	1	1	JCK-3
gugccaugaacuugCuccaga	1	1	JCK-1
gugccCugaacuugauccaga	4	1	JCK-1
gugUcaugaacuugauccaga	2	1	JCK-3
gugccCugaacuugauccaga	2	1	JCK-3
gugccGugaacuugauccaga	1	1	JCK-1
gugccaugaacuugauccCga	1	1	LT-3
gugccaugCacuugauccaga	2	1	LT-2
gugccaugCacuugauccaga	1	1	JCK-2
gugccaugaacuugauccagaC	4	1	LT-1
gugccaugaacuugauccagaC	3	1	LT-3
gugccaugaacuugauccagaU	1	1	LT-1
Uugccaugaacuugauccagaa	4	1	JCK-3
gugccaugaacuugauccagaU	2	1	JCK-3
gugccaugaacuugauccagaC	2	1	JCK-1
gugccaugaacuugauccagaa	5	0	LT-3
Uugccaugaacuugauccagaa	1	1	JCK-1
gugccaugaacuugauccagaa	2	0	LT-1
gugccaugaacuugauccagaa	8	0	JCK-2
gugccaugaacuugauccagaa	11	0	LCK-3
gugccaugaacuugauccagaU	3	1	JCK-1
Uugccaugaacuugauccagaa	2	1	JCK-2
gugccaugaacuugauccaAaa	1	1	JCK-2
gugccaugaacuugauccagaC	4	1	LT-2
gugccaugaacuugauccagaa	4	0	JCK-1
gugccaugaacuugauccagaU	3	1	JCK-2
gugccaugaacuugauccagaa	9	0	JCK-3
gugccaugaacuugauccagaC	2	1	JCK-2
gugccaugaacuugauccagaaA	3	1	JCK-1
gugccaugaacuugauccagaaA	8	1	JCK-2
gugccaugaacuugauccagaaA	3	1	JCK-3
gugccaugaacuugauccagaagguU	2	1	JCK-3
.ugccaugaacuugaucca	6	0	LT-3
.ugccaugaacuugaucca	8	0	LCK-3

Mature Star

```
uccaucuuguucguucacccucuggaucuuguucgauggcacuccccuuguauccaaucucucucucucucucucucucucucucucucucauauggugcgaguggaagugccaugaacuugauccagaaggugcucucgaacaaguuacaccuacuuaauaucuacuucuucuuucuccauuacuauuauuaaugaugguuucuuuuuucugauucucauuuuuaauuuauuuauuuguuuuuguguuug
```

ugccaugaacuugaCcca	1	1	LT-3
ugccaugaacuugaucca	1	0	JCK-3
ugccaugaacuugaucca	7	0	LT-2
ugccaugaacuugaucca	1	0	JCK-1
ugccaugaacuugaucca	7	0	JCK-2
ugccaugaacuugaucca	1	0	LT-1
ugccaugaacuugauccag	74	0	LCK-3
ugccaugCacuugauccag	1	1	JCK-3
ugccaugaacuugauccag	101	0	JCK-1
ugccaugaacuugauccCg	1	1	LT-2
ugccaugaacuugauccag	102	0	JCK-3
ugccaugaacuugauccaA	1	1	JCK-1
ugccaugaacuugauccaU	1	1	LT-3
ugccaugaacuugauccag	161	0	LT-1
ugccaugCacuugauccag	1	1	LT-1
ugccaugaacuugauccag	167	0	JCK-2
ugccaugaacuugauccaA	1	1	JCK-2
ugccaCgaacuugauccag	1	1	JCK-2
ugccaugaacuugauccag	43	0	JT-3
ugccaugaacuugauccag	26	0	JT-2
ugccaugaacuugauccag	9	0	JT-1
ugccauCaacuugauccag	1	1	JCK-1
ugccaugaCcuugauccag	1	1	LT-1
ugccaugaacuugauccaU	1	1	JCK-1
ugccaugaCcuugauccag	1	1	LT-3
ugccaugaCcuugauccag	1	1	LT-2
ugccaugaacuugauccCg	1	1	JCK-2
Cgccaugaacuugauccag	1	1	JCK-1
ugccaugaacuugauccag	259	0	LT-3
Cgccaugaacuugauccag	2	1	JCK-3
ugccaugaacuugauccaA	2	1	JCK-3
ugccaugaacuugauccag	114	0	LT-2
uNccaugaacuugauccag	1	1	JCK-1
Cgccaugaacuugauccag	1	1	JCK-2
ugccaugaacuugauccagG	1	1	JCK-2
ugccaugaacuugauccagC	2	1	JCK-3
ugccaugaacuugauccaga	184	0	LCK-3
ugccaugCacuugauccaga	1	1	LCK-3
ugccaugaacuugauccagU	7	1	JCK-3
uNccaugaacuugauccaga	2	1	JCK-1
ugccaugaacuugauccaga	1134	0	JCK-3
ugccaugCacuugauccaga	1	1	JT-1
ugccaugaacuugauccagU	5	1	JCK-2
ugccaugaacuugauccagG	1	1	JCK-1
ugccaugaacuugauccaga	848	0	JCK-1
ugccGugaacuugauccaga	1	1	LT-1
ugccaugaacuugauccagG	1	1	LT-2
ugcAaugaacuugauccaga	1	1	JCK-2
ugccaugaacuugauccaga	60	0	LCK-1
ugccaugaacuNgauccaga	1	1	JCK-1
uAccaugaacuugauccaga	1	1	JCK-2
ugccaugaCcuugauccaga	1	1	LT-1
ugccaugaCcuugauccaga	1	1	JCK-2
ugccaugaacuugCuccaga	1	1	LCK-3
ugccauAaacuugauccaga	1	1	LT-3
ugccaugaacuugauccaNa	1	1	JCK-1
uAccaugaacuugauccaga	2	1	LT-1
uUccaugaacuugauccaga	1	1	JCK-3
ugcAaugaacuugauccaga	1	1	JCK-1
ugccaugaacuugauccCga	2	1	JCK-1
ugccaugaacuugauccagN	1	1	JCK-1
ugccCugaacuugauccaga	1	1	LT-2
ugccaugaacuugauccaga	290	0	LT-2
ugccaugaacuugauccagU	3	1	LT-1
ugccaugaacuugaCccaga	1	1	JCK-3
ugccaugaacuugauccaga	476	0	LT-1
ugccaugaacuugauccaAa	1	1	JCK-3
ugccaugCacuugauccaga	1	1	LT-2
ugccaugaacuugauccaga	86	0	LCK-2
ugccaugaacuugauccCga	3	1	JCK-2

```
                      Mature                                                                      Star
uccaucuuguucguucacccucuggaucuuguucgauggcacuccccuuguauccaaucucucucucucucucucucucucucucucucauauggugcgaguggaagugccaugaacuugauccagaaggugcucucgaacaaguuacaccuacuuaauaucuacuucuucuuucuccauuacuauuauuaaugaugguuucuuuuuucugauucucauuuuaauuuauuuauuuguuuuuguguuug
...........................................................................................................ugccCugaacuugauccaga........................................................................................................................   1     1   LT-3
...........................................................................................................ugccaugaacuugauccaga........................................................................................................................   6     0   JT-2
...........................................................................................................ugccaugaacuugCuccaga........................................................................................................................   1     1   LT-3
...........................................................................................................ugccaugaacuugauccagC........................................................................................................................   3     1   JCK-2
...........................................................................................................uNccaugaacuugauccaga........................................................................................................................   2     1   JCK-3
...........................................................................................................ugccaugaacuugauccaga........................................................................................................................   12    0   JT-3
...........................................................................................................ugccaugaacuugauccaga........................................................................................................................   1507  0   JCK-2
...........................................................................................................ugccaugaacuuUauccaga........................................................................................................................   1     1   JCK-2
...........................................................................................................ugccaugaacuugauccaga........................................................................................................................   93    0   JT-1
...........................................................................................................ugccaugGacuugauccaga........................................................................................................................   1     1   JCK-2
...........................................................................................................ugccaugaacuugauccagC........................................................................................................................   4     1   LT-3
...........................................................................................................Cgccaugaacuugauccaga........................................................................................................................   1     1   LCK-3
...........................................................................................................ugccCugaacuugauccaga........................................................................................................................   1     1   JCK-2
...........................................................................................................ugccaugaacuugauNcaga........................................................................................................................   1     1   JCK-3
...........................................................................................................ugccaugaCcuugauccaga........................................................................................................................   1     1   JCK-1
...........................................................................................................uAccaugaacuugauccaga........................................................................................................................   1     1   LCK-3
...........................................................................................................ugcGaugaacuugauccaga........................................................................................................................   2     1   JCK-1
...........................................................................................................ugccaugaCcuugauccaga........................................................................................................................   4     1   JCK-3
...........................................................................................................ugccaugaacuuUauccaga........................................................................................................................   1     1   JCK-3
...........................................................................................................ugccaugaacuugCuccaga........................................................................................................................   1     1   JT-1
...........................................................................................................ugccaugaacuugauccagU........................................................................................................................   4     1   LT-3
...........................................................................................................ugccaugaacuCgauccaga........................................................................................................................   1     1   JCK-3
...........................................................................................................ugNcaugaacuugauccaga........................................................................................................................   1     1   LT-2
...........................................................................................................ugccaugaacuugauccagU........................................................................................................................   1     1   JCK-1
...........................................................................................................ugccaugaacuugauccaga........................................................................................................................   657   0   LT-3
...........................................................................................................ugccaugCacuugauccaga........................................................................................................................   1     1   LT-3
...........................................................................................................ugccCugaacuugauccaga........................................................................................................................   4     1   JCK-3
...........................................................................................................ugccaugaacuugauccagU........................................................................................................................   9     1   LT-2
...........................................................................................................ugccCugaacuugauccaga........................................................................................................................   1     1   LT-1
...........................................................................................................ugccaugaacuugauccagaU.......................................................................................................................   5     1   LCK-3
...........................................................................................................ugccaugaacuugauccagaC.......................................................................................................................   3     1   LT-1
...........................................................................................................ugccaugaacuugauccagaa.......................................................................................................................   4     0   LT-2
...........................................................................................................ugccaugaacuugauccagaU.......................................................................................................................   2     1   JT-3
...........................................................................................................ugccaugaacuugauccagaU.......................................................................................................................   1     1   JCK-1
...........................................................................................................ugccaugaacuugauccagaa.......................................................................................................................   5     0   JCK-1
...........................................................................................................ugccaugaacuugauccagaU.......................................................................................................................   2     1   JCK-2
...........................................................................................................ugccaugaacuugauccagaU.......................................................................................................................   9     1   LT-3
...........................................................................................................ugccaugaacuugauccagaU.......................................................................................................................   5     1   LT-2
...........................................................................................................ugccaugaacuugauccagaa.......................................................................................................................   1     0   JCK-2
...........................................................................................................ugccaugaacuugauccagaa.......................................................................................................................   3     0   JCK-3
...........................................................................................................ugccaugaacuugauccagaU.......................................................................................................................   10    1   LT-1
...........................................................................................................ugccaugaacuugauccagaC.......................................................................................................................   5     1   LT-3
...........................................................................................................ugccaugaacuugauccagaag......................................................................................................................   1     0   JCK-2
...........................................................................................................ugccaugaacuugauccagaag......................................................................................................................   2     0   JCK-3
...........................................................................................................ugccaugaacuugauccagaagg.....................................................................................................................   5     0   LT-3
...........................................................................................................ugccaugaacuugauccagaagg.....................................................................................................................   1     0   JCK-1
...........................................................................................................ugccaugaacuugauccagaagg.....................................................................................................................   3     0   JCK-3
...........................................................................................................ugccaugaacuugauccagaagg.....................................................................................................................   1     0   JCK-2
............................................................................................................gccaugaacuugauccag.........................................................................................................................   1     0   JCK-2
............................................................................................................gccaugaacuugauccaga........................................................................................................................   1     0   JCK-1
............................................................................................................gccaugaacuugauccaga........................................................................................................................   1     0   JCK-3
.............................................................................................................ccaugaacuugauccagU........................................................................................................................   1     1   JCK-3
.............................................................................................................ccaugaacuugauccaga........................................................................................................................   2     0   LT-3
.............................................................................................................ccaugaacuugauccaga........................................................................................................................   1     0   JCK-2
.............................................................................................................ccaugaacuugauccagU........................................................................................................................   4     1   LCK-3
.............................................................................................................ccaugaacuugauccagU........................................................................................................................   2     1   JCK-1
.............................................................................................................ccaugaacuugauccagaaggu....................................................................................................................   1     0   LT-3
..................................................................................................................aacuugauccagaaggugcucu...............................................................................................................   1     0   JCK-2
..................................................................................................................aacuugauccagaaggugcucu...............................................................................................................   1     0   JCK-1
...................................................................................................................acuugauccagaaggugcucucga............................................................................................................   1     0   LT-2
.........................................................................................................................uccagaaggugcucucgaacaaguuac...................................................................................................   1     0   LT-2
...........................................................................................................................cagaaggugcucucgaacaagu......................................................................................................   2     0   JCK-1
...........................................................................................................................cagaaggugcucucgaacaagu......................................................................................................   4     0   JCK-2
...........................................................................................................................cagaaggugcucucgaacaagu......................................................................................................   3     0   LT-3
...........................................................................................................................cagaaggugcucucgaacaagu......................................................................................................   7     0   LCK-3
...........................................................................................................................cagaaggugcucucgaacaagu......................................................................................................   4     0   JCK-3
...........................................................................................................................cagaaggugcucucgaacaagu......................................................................................................   2     0   LT-1
............................................................................................................................agaaggugcucucgaacaagu......................................................................................................   1     0   LT-1
............................................................................................................................agaaggugcucucgaacaNgu......................................................................................................   1     1   JCK-1
............................................................................................................................agaaggugcucucgaacaagu......................................................................................................   1     0   JCK-3
```

```
                    Mature                                                                           Star
uccaucuuguucguucacccucuggaucuuguucgauggcacuccccuuguauccaaucucucucucucucucucucucucucucucucauauggugcgaguggaagugccaugaacuugauccagaaggugcucucgaacaaguuacaccuacuuaauaucuacuucuucuuucuccauuacuauuauuaaugaugguuucuuuuucugauucucauuuuuaauuuauuuauuuguuuuuguguuug
............................................................................................................................agaaggugcucucgaacaagu.......................................................................................................   7   0   LT-3
............................................................................................................................agaaggugcucucgaacaagu.......................................................................................................   3   0   JCK-2
............................................................................................................................agaaggugcucucgaacaagu.......................................................................................................   6   0   LCK-3
............................................................................................................................agaaggugcucucgaacaaguu......................................................................................................   3   0   JCK-1
............................................................................................................................agaaggugcucucgaacaaguu......................................................................................................   1   0   LT-1
............................................................................................................................agaaggugcucucgaacaaguu......................................................................................................   1   0   JCK-3
............................................................................................................................agaaggugcucucgaacaaguu......................................................................................................   2   0   LT-3
............................................................................................................................agaaggugcucucgaacaaguuaca...................................................................................................   1   0   LT-3
.............................................................................................................................gaaggugcucucgaacaaguua.....................................................................................................   3   0   LT-1
.............................................................................................................................gaaggugcucucgaacaaguuac....................................................................................................   2   0   JCK-3
.............................................................................................................................gaaggugcucucgaacaaguuac....................................................................................................   1   0   JCK-2
...............................................................................................................................aggugcucucgaacaagu.......................................................................................................   1   0   LT-3
...............................................................................................................................aggugcucucgaacaaguA......................................................................................................   1   1   LT-1
...............................................................................................................................aggugcucucgaacaaguuacC...................................................................................................   1   1   LT-3
...............................................................................................................................aggugcucucgaacaaguuaca...................................................................................................   1   0   LT-2
```
